# Senomorphic Small Extracellular Vesicles Delivered by a Tissue‐Adhesive α‐Lipoic‐Acid Hydrogel Enable Immuno‐Rejuvenation for Bone‐Tendon Interface Regeneration

**DOI:** 10.1002/advs.202524366

**Published:** 2026-04-07

**Authors:** Lingzhi Kong, Wei Song, Wencai Liu, Hui Xu, Yuhao Yu, Xinyue Yang, Haiyan Li, Yanlun Zhu, Yaohua He

**Affiliations:** ^1^ Department of Sports Medicine Shanghai Sixth People's Hospital Affiliated to Shanghai Jiao Tong University School of Medicine Shanghai P. R. China; ^2^ Biomedical Engineering Department, School of Engineering STEM College, RMIT University Melbourne Victoria Australia; ^3^ State Key Laboratory of High Performance Ceramics Shanghai Institute of Ceramics Chinese Academy of Sciences Shanghai P. R. China; ^4^ Department of Orthopedic Surgery Jinshan District Central Hospital affiliated to Shanghai University of Medicine & Health Sciences Jinshan Branch of Shanghai Sixth People's Hospital Shanghai P. R. China

**Keywords:** BMSC senescence, bone‐tendon interface regeneration, cGAS‐STING/NF‐κB signaling, senomorphic extracellular vesicles, α‐Lipoic acid hydrogel

## Abstract

Chronic inflammation‐driven bone loss in aging compromises bone regeneration and further impairs the bone‐tendon interface (BTI). However, the cellular mechanisms by which inflammation exacerbates cellular senescence and consequently disrupts BTI healing remain unclear. Here, we identify M1 macrophage‐mediated inflammation as a key driver of bone marrow‐derived mesenchymal stem cells (BMSCs) senescence and bone microstructural deterioration. This senescence‐associated decline in BMSCs ultimately compromises osteogenesis and delays BTI repair. To counteract these effects, we engineered a senomorphic and immunomodulatory platform by incorporating quercetin‐primed senomorphic small extracellular vesicles (Sm‐sEV) into a tissue‐adhesive α‐lipoic acid hydrogel (αLA‐Gel) for sustained local delivery. The composite material modulates the inflammatory‐senescent microenvironment by attenuating M1 macrophage‐driven inflammation and enhancing BMSC resilience to inflammation‐exacerbated senescence. Mechanistic analyses revealed that Sm‐sEV/αLA‐Gel suppresses cGAS‐STING‐NF‐κB signaling, thereby reducing inflammation and improving BMSC resistance to senescence. In an osteoporotic rat rotator cuff repair model, Sm‐sEV/αLA‐Gel enhanced bone formation and fibrocartilage maturation, thereby promoting superior BTI integration and mechanical strength. Together, these findings identify inflammation‐exacerbated BMSC senescence as a key pathological driver and demonstrate that dual regulation of inflammation and stem cell resilience enables robust regeneration of bone and the BTI under osteoporotic conditions.

## Graphical abstract

This work identifies M1 macrophage‐mediated inflammation as a key driver of BMSC senescence and bone microstructural deterioration at the enthesis. A tissue‐adhesive hydrogel delivering senomorphic small extracellular vesicles suppresses this inflammatory‐senescent cascade and enhances BMSC resilience by inhibiting the cGAS‐STING‐NF‐κB pathway. In an osteoporotic rat model, this dual immunomodulatory and senomorphic strategy accelerates bone‐tendon interface regeneration.

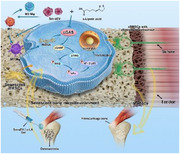

## 1 Introduction

The regenerative capacity of bone and the bone‐tendon interface (BTI) declines with age, making the restoration of complex musculoskeletal interfaces a persistent clinical challenge [1]. Aging is invariably accompanied by a state of chronic, low‐grade inflammation that accelerates cellular senescence and disturbs bone remodeling, thereby weakening skeletal integrity and delaying repair [2]. Rotator cuff tear is a common manifestation of this degenerative process, with its prevalence rising sharply from 9.7% in individuals under 20 years to over 60% in those aged above 80 [3]. This age‐related vulnerability is closely linked to osteoporosis, a chronic inflammatory bone disorder that affects about one‐third of women and one‐fifth of men over 50 years old worldwide [4]. In osteoporosis, sustained inflammation accelerates bone resorption and structural fragility, and the resulting degeneration at the BTI severely compromises regenerative healing in elderly patients [5, 6, 7].

At the cellular level, this degenerative environment profoundly alters the behavior of resident immune and stem cells, further limiting the intrinsic repair potential. Notably, bone marrow‐derived mesenchymal stem cells (BMSCs) derived from osteoporotic individuals display a baseline senescent phenotype, characterized by reduced proliferative and osteogenic capacity and increased expression of senescence‐associated markers [8]. Meanwhile, macrophages, the central regulators of the inflammatory microenvironment, lose their normal capacity to resolve inflammation, leading to prolonged immune activation and delayed repair. This macrophage‐driven inflammatory state amplifies BMSC senescence, disrupts matrix remodeling, and ultimately undermines bone regeneration [9]. Yet, the molecular mechanisms linking inflammaging to impaired BTI regeneration remain poorly understood, and effective strategies to reverse this degenerative microenvironment are still lacking.

Although various biomaterial‐based and pharmacological strategies can effectively suppress inflammation and promote repair in young or non‐senescent models, their efficacy is markedly limited in aging organisms, where chronic inflammation and cellular senescence form a self‐perpetuating cycle that amplifies tissue degeneration and impairs regeneration [10]. In osteoporotic or aged environments, conventional biomaterials lose much of their immunoregulatory efficacy because impaired macrophage polarization sustains inflammation and limits osteogenesis [11]. A more effective strategy may involve simultaneously modulating inflammation and alleviating inflammation‐induced stem cell senescence to enhance BTI healing [12, 13].

Small extracellular vesicles (sEV) have attracted increasing attention for musculoskeletal repair because of their excellent biocompatibility and ability to modulate immune responses and promote osteogenesis [14]. Unlike conventional materials, sEV mediate active intercellular signaling by delivering functional proteins and RNAs that reprogram recipient‐cell behavior and tissue responses. However, their therapeutic efficacy is limited under chronic inflammatory and senescent conditions [15, 16]. Enhancing their senomorphic and anti‐inflammatory activity may therefore help overcome these age‐related barriers to regeneration. One feasible strategy to achieve this is to functionally reprogram sEV through pharmacological stimulation [17]. For example, succinate‐preconditioned exosomes enriched in bioenergetic metabolites have been shown to elevate adenosine triphosphate levels and promote cartilage regeneration by reprogramming cellular metabolism [18]. Pharmacological priming augments the therapeutic potential of sEV by integrating its innate signaling and delivery capabilities with the functional activity of small molecules. Quercetin, a natural flavonoid with well‐documented anti‐inflammatory and senolytic effects, offers a promising means to reprogram sEV bioactivity [19]. Building on this rationale, we hypothesized that preconditioning donor cells with quercetin would generate senomorphic small extracellular vesicles (Sm‐sEV) with enhanced immunomodulatory and secondary enhancement of osteogenesis.

A major challenge in EV‐based therapy is their rapid clearance and limited retention at the injury site, which significantly restricts therapeutic efficacy [20]. α‐Lipoic acid (αLA), a small‐molecule antioxidant capable of forming dynamic disulfide bonds, has been used to construct adhesive and self‐healing hydrogels for controlled release and vesicle delivery [21]. Building on these features, we developed an αLA‐hydrogel (αLA‐Gel), a novel mucus‐like matrix with stronger wet adhesion and flexibility than conventional hydrogels [22]. We hypothesize that this adhesive interface could prolong Sm‐sEV retention and provide additional immunoregulatory support, thereby improving BTI healing under osteoporotic conditions [23].

**SCHEME 1:**
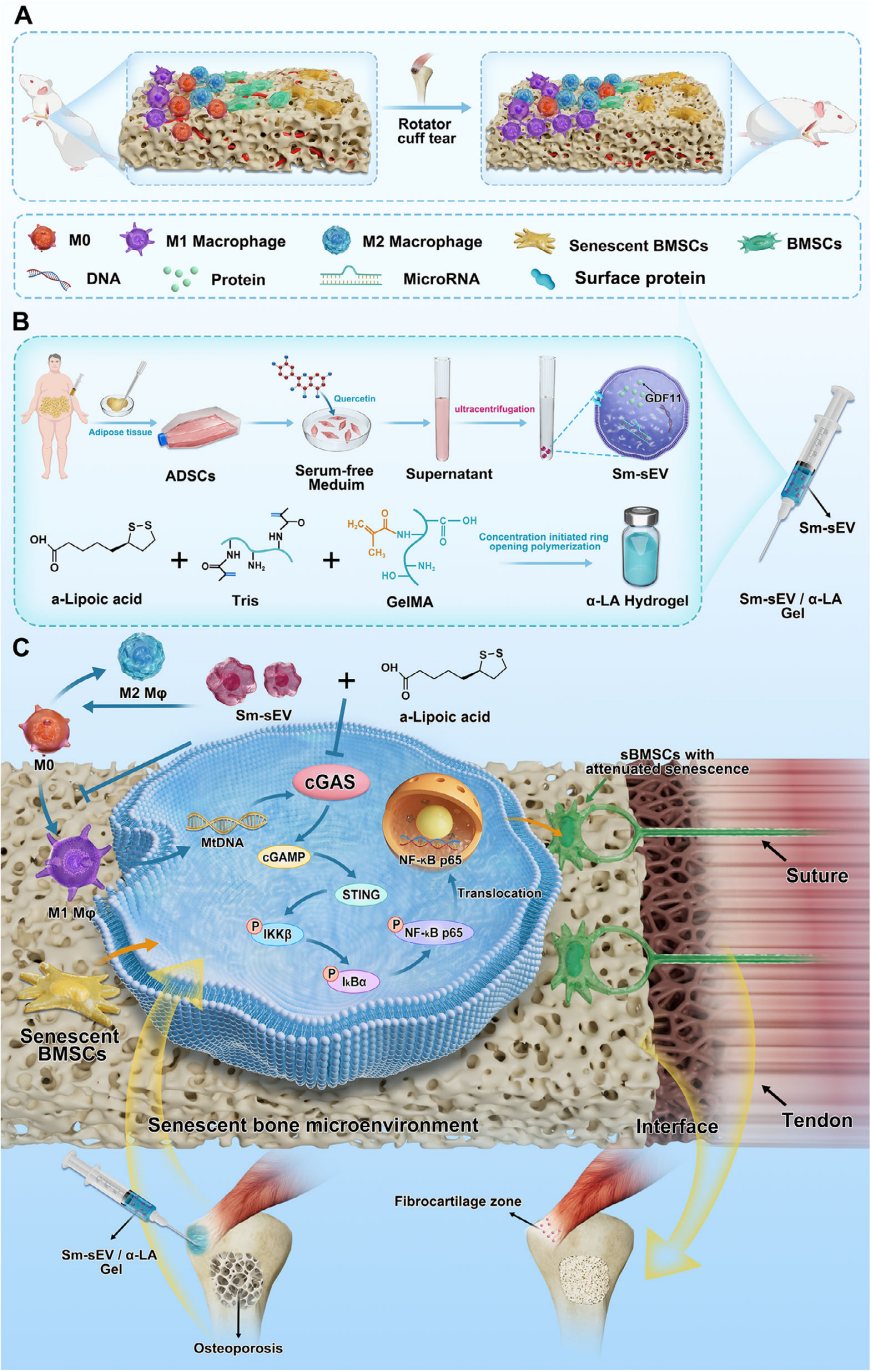
Schematic illustration of the design and mechanism of Sm‐sEV/αLA‐Gel for reprogramming the aged osteoporotic microenvironment and promoting bone‐tendon healing. (A) Pathological features of the osteoporotic BTI following rotator cuff injury. The aged microenvironment is characterized by sustained M1 macrophage infiltration, accumulation of senescent BMSCs, and disrupted immune‐regenerative balance. (B) Preparation process of the Sm‐sEV/αLA‐Gel system. Quercetin‐preconditioned ADSCs secrete Sm‐sEV, which are combined with αLA‐based hydrogel generated through dynamic crosslinking between αLA and GelMA. The resulting Sm‐sEV/αLA‐Gel composite hydrogel enables minimally invasive injection and sustained release at the injury site. (C) Proposed mechanism of action. Sm‐sEV/αLA‐Gel reprograms the osteoporotic microenvironment by modulating the cGAS‐STING‐NF‐κB pathway, thereby suppressing inflammation and cellular senescence. This integrated regulation restores a pro‐regenerative niche and promotes synchronized bone‐fibrocartilage‐tendon interfacial regeneration under osteoporotic conditions.

In this study, we first delineated how an inflammatory microenvironment drives BMSC senescence and impairs BTI regeneration in osteoporotic conditions. Building on this mechanistic insight, we engineered Sm‐sEV to enhance the intrinsic immunomodulatory capacity of native sEV, aiming to more effectively regulate macrophage polarization and alleviate inflammation‐exacerbated BMSC senescence. We then incorporated Sm‐sEV into a bioadhesive αLA‐Gel (Sm‐sEV/αLA‐Gel) to prolong local retention and potentiate therapeutic efficacy. Finally, we systematically evaluated its anti‐inflammatory and anti‐senescence effects in vitro and regenerative potential in an osteoporotic rat rotator cuff repair model (Scheme 1).

## 2 Results

### 2.1 Inflammation‐driven BMSC Senescence Exacerbates Bone Microenvironmental Degeneration After Rotator Cuff Injury

**FIGURE 1:**
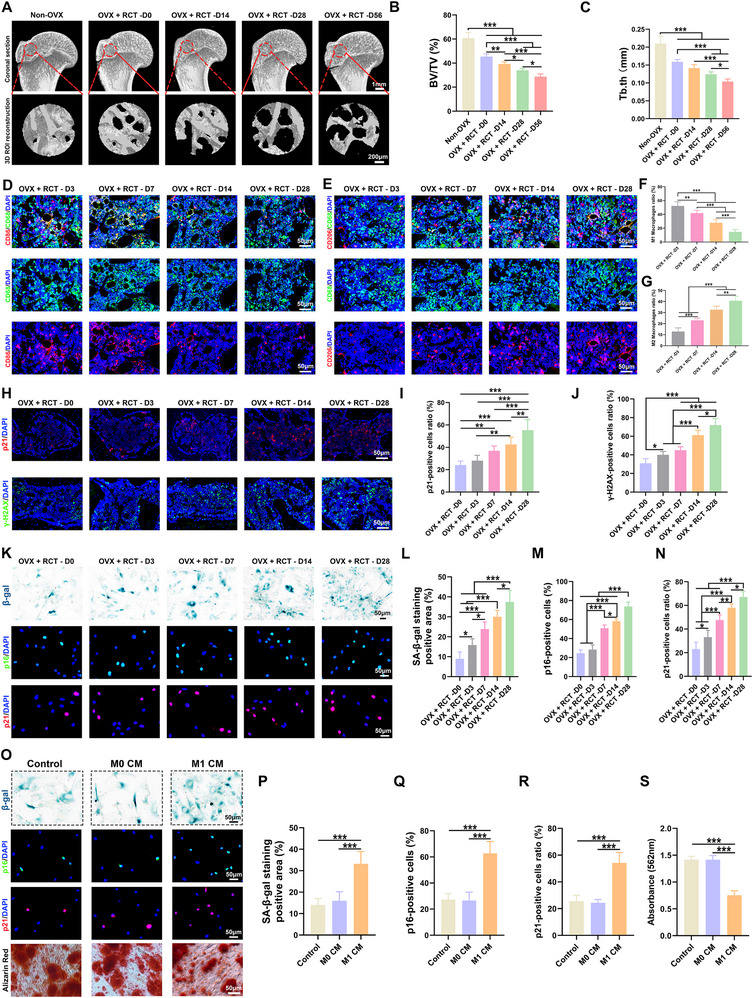
Characterization of inflammation‐exacerbated BMSC senescence and bone microenvironmental degeneration following rotator cuff injury in osteoporotic rats. (A) Representative micro‐CT 3D reconstructions of the proximal humerus showing progressive bone loss and trabecular deterioration in OVX rats at the indicated post‐injury time points. (B,C) Quantitative analysis of BV/TV and Tb.Th. (D–G) Immunofluorescence staining for CD68^+^CD86^+^ (M1) and CD68^+^CD206^+^ (M2) macrophages demonstrates the temporal pattern of macrophage infiltration and transition. (H–J) Immunostaining for p21 and γ‐H2AX showing time‐dependent activation of senescence markers in bone tissue. (K–N) SA‐β‐gal staining and quantification of p16^+^ and p21^+^ cells in BMSCs isolated from different post‐injury time points. (O–S) In vitro assays of BMSCs stimulated with M0 or M1 CM showing differences in senescence marker expression and osteogenic differentiation. n = 6. ^*^ *p* < 0.05, ^**^ *p* < 0.01, ^***^ *p* < 0.001.

To investigate the pathological impact of inflammation in an estrogen‐deficient, aging‐like skeletal microenvironment, we established a rotator cuff tear (RCT) model in ovariectomized (OVX) rats, with non‐OVX animals serving as controls. Micro‐computed tomography (micro‐CT) analysis revealed a progressive loss of bone mass and deterioration of trabecular structure in the OVX+RCT model. Bone volume/total volume (BV/TV) decreased gradually from Non‐OVX and OVX+RCT‐D0 to D14, D28, and D56 (representing 0, 14, 28, and 56 days post‐OVX and RCT), showing a time‐dependent reduction. Trabecular thickness (Tb. Th) and trabecular number (Tb. N) displayed a similar downward trend but without significant differences between D0 and D14 or between D14 and D28. In contrast, Trabecular separation (Tb. Sp) remained low in the Non‐OVX and D0 groups, increased at D28, and peaked at D56, where values were significantly higher than in Non‐OVX, D0, and D14. These findings indicate progressive trabecular rarefaction and structural deterioration in osteoporotic bone after rotator cuff injury (Figure 1A–C; Figure S1).

Immunofluorescence staining showed that CD68^+^CD86^+^ (M1) macrophages were most abundant at day 3, remained elevated at day 7, and progressively decreased at days 14 and 28 (Figure 1D,F). In contrast, CD68^+^CD206^+^ (M2) macrophages were minimal at days 3 and 7, increased at day 14, and peaked at day 28 (Figure 1E,G). These temporal dynamics indicate a delayed inflammatory‐to‐reparative transition within the osteoporotic rotator cuff injury microenvironment, characterized by sustained early inflammation and a postponed reparative shift.

Consistent with the inflammatory dynamics, immunofluorescence staining for p21 and phosphorylated H2A histone family member X (γ‐H2AX) demonstrated early activation of senescence signals at day 3 post‐injury, with a progressive increase and a peak at day 28 (Figure 1H–J), suggesting that early inflammation may contribute to the induction of cellular senescence. To further evaluate the relationship between inflammation and stem cell senescence, BMSCs were isolated from the proximal humeral bone marrow at different time points after injury and cultured ex vivo. Senescence‐associated β‐galactosidase (SA‐β‐gal) staining and quantification of p16^+^ or p21^+^ cells revealed a time‐dependent increase in BMSC senescence (Figure 1K–N), which closely mirrored the in vivo inflammatory pattern, implicating inflammation as a potential driver of stem cell aging.

To validate this hypothesis, conditioned medium from M1 macrophages (M1 CM) was used to stimulate BMSCs in vitro. Considering that BMSCs from aged or osteoporotic hosts exhibit a baseline senescent phenotype, passage 10 BMSCs were selected as a replicative senescence model to better mimic the aged microenvironment, and their senescent phenotype was validated by SA‐β‐gal staining and canonical senescence markers, including p16 and p21 [24]. M1 CM treatment markedly enhanced SA‐β‐gal activity and p16/p21 expression in BMSCs and significantly suppressed their osteogenic mineralization capacity, whereas M0 CM showed no obvious effect (Figure 1O–S). Taken together, these results indicate that an inflammatory microenvironment, particularly M1 macrophage‐derived factors, drives premature BMSC senescence and compromises their osteogenic capacity, ultimately forming an unfavorable niche for regeneration after osteoporotic rotator cuff injury.

### 2.2 Characterization and Immunomodulatory and Senomorphic Effects of Sm‐sEV

**FIGURE 2:**
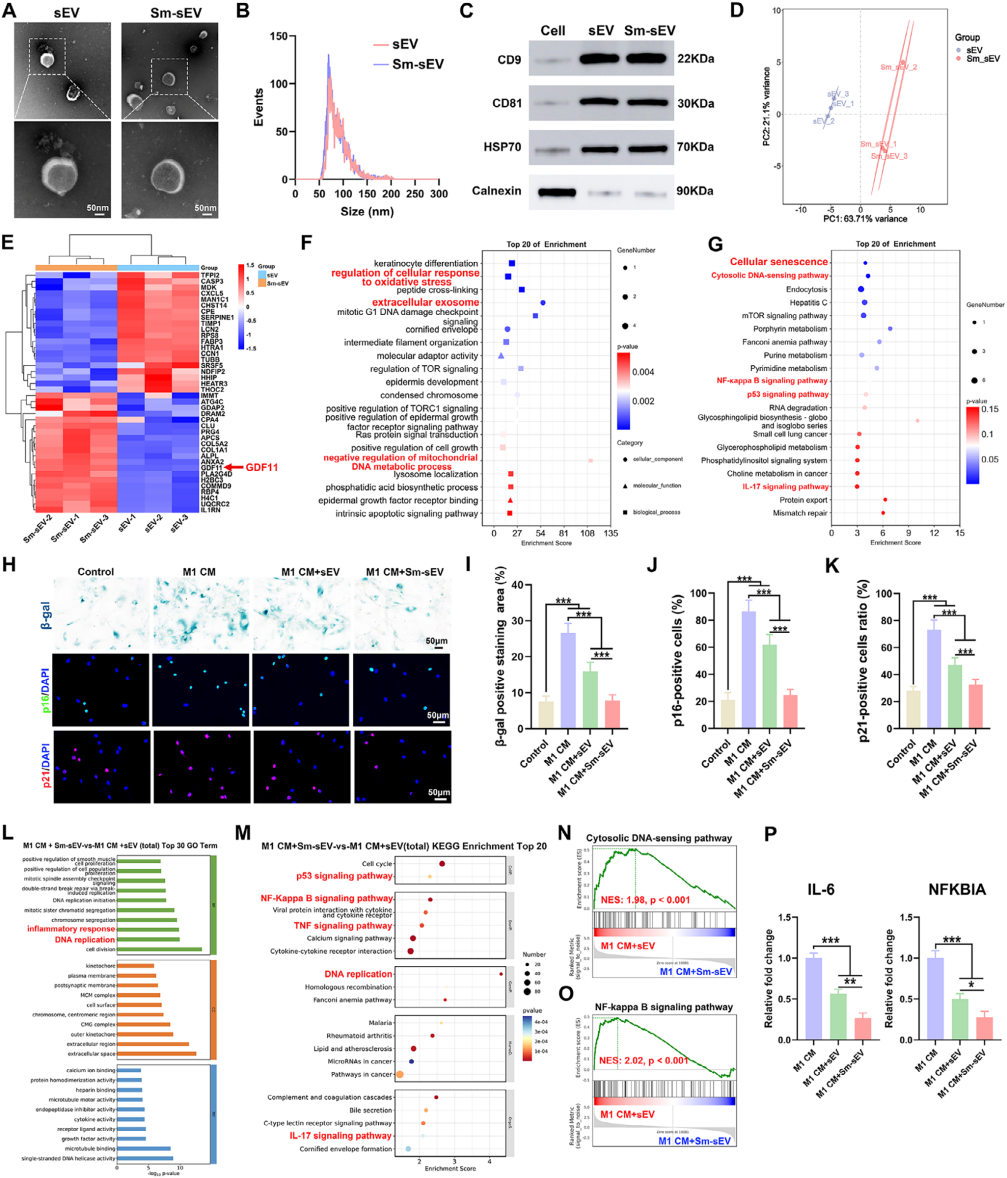
Characterization of Sm‐sEV and their role in mitigating M1 CM‐exacerbated senescence in BMSCs. (A–C) TEM, nanoparticle tracking analysis, and western blotting confirmed the morphology, size distribution, and purity of sEV and Sm‐sEV. (D) PCA score plot showing distinct proteomic profiles between sEV and Sm‐sEV. (E–G) Heatmap, GO, and KEGG analyses. (H–K) SA‐β‐gal staining and immunofluorescence analyses for p16 and p21, along with their semi‐quantitative evaluation. (L–O) GO, KEGG, and GSEA analyses between M1 CM+sEV and M1 CM+Sm‐sEV. (P) RT‐qPCR validation of NF‐κB‐related gene expression in BMSCs treated with M1 CM, sEV, or Sm‐sEV. n = 3. ^*^ *p* < 0.05, ^**^ *p* < 0.01, ^***^ *p* < 0.001.

Small extracellular vesicles were isolated from adipose‐derived stem cells (ADSCs) and functionally engineered through quercetin stimulation to generate Sm‐sEV. Transmission electron microscopy (TEM) and nanoparticle tracking analysis revealed a uniform spherical morphology with a bilayer membrane and a diameter of approximately 50–150 nm (Figure 2A–C). Western blotting confirmed the positive expression of CD9, CD81, and HSP70, while calnexin was undetectable, indicating high vesicle purity. Principal component analysis (PCA) demonstrated a clear separation between sEV and Sm‐sEV, suggesting significant proteomic alterations following quercetin stimulation (Figure 2D). Comparative proteomic profiling identified 114 differentially expressed proteins, including 58 upregulated and 56 downregulated proteins (Figure S2).

To elucidate the molecular basis of their enhanced bioactivity, we performed proteomic and functional enrichment analyses. Heatmap clustering demonstrated a distinct protein expression pattern between the two groups. Notably, several proteins associated with cellular senescence were upregulated in Sm‐sEV, including GDF11, Interleukin 1 receptor antagonist (IL1RN) and Clusterin (CLU) (Figure 2E). GDF11 is a rejuvenation factor known to counteract age‐related functional decline [25]. GO analysis indicated enrichment in oxidative stress response, extracellular vesicle function, and mitochondrial metabolism (Figure 2F). KEGG analysis further highlighted significant enrichment in cellular senescence, cytosolic DNA sensing, NF‐κB, p53, and IL‐17 signaling pathways, consistent with the observed anti‐inflammatory and anti‐senescence effects (Figure 2G).

To assess the immunomodulatory capacity of Sm‐sEV, we examined its impact on macrophage polarization. RT‐qPCR revealed that M1 CM stimulation significantly increased IL‐1β, TNF‐α, and IL‐6 expression, while both sEV and Sm‐sEV reduced their levels, with Sm‐sEV showing a more pronounced suppressive effect. In contrast, Sm‐sEV markedly enhanced the expression of Arg‐1, IL‐1ra, and IL‐10, indicating promotion of M2 polarization. Immunofluorescence staining corroborated these results, showing decreased iNOS signal and enhanced CD206 expression after Sm‐sEV treatment compared to sEV. These data indicate that Sm‐sEV exerts stronger immunomodulatory effects than native sEV, effectively suppressing M1‐type inflammation while promoting M2 polarization (Figure S3).

We next evaluated the anti‐senescence effect of Sm‐sEV under inflammatory stress. Treatment of BMSCs with M1 CM significantly increased SA‐β‐gal staining, while both sEV and Sm‐sEV reduced SA‐β‐gal activity, with Sm‐sEV showing the most pronounced inhibition (Figure 2H‐I). Similarly, M1 CM strongly upregulated the expression of senescence markers p16 and p21, whereas both sEV and Sm‐sEV attenuated their levels, and Sm‐sEV exhibited significantly greater suppression (Figure 2H,J,K). Consistently, RT‐qPCR analysis revealed similar trends in the mRNA levels of p16, p21, and γ‐H2AX (Figure S4). Compared with the control group, M1 CM markedly increased the transcription of these senescence‐associated genes, while sEV partially reversed this induction. Notably, Sm‐sEV treatment resulted in the most significant downregulation, indicating a stronger protective effect against inflammation‐aggravated senescence. Together, these results demonstrate that Sm‐sEV more effectively suppresses senescence at both the phenotypic and transcriptional levels compared with unmodified sEV.

To further dissect the underlying molecular mechanisms, we performed transcriptomic sequencing and RT‐qPCR validation on M1 CM‐aggravated senescent BMSCs treated with sEV or Sm‐sEV. GO enrichment analysis revealed that Sm‐sEV‐responsive genes were primarily enriched in inflammatory response, cell cycle regulation, and DNA replication pathways (Figure 2L). KEGG analysis further highlighted NF‐κB, TNF, p53, and IL‐17 signaling pathways (Figure 2M), while GSEA demonstrated significant downregulation of the cytosolic DNA‐sensing, NF‐κB signaling pathways, and TNF signaling pathway in the Sm‐sEV group compared to sEV (Figure 2N–O; Figure S5). RT‐qPCR analysis showed that Sm‐sEV markedly reduced the expression of NF‐κB downstream genes, including IL‐6, CCL2, NFKBIA, IL‐1β, PTGS2, and TNF, and its inhibitory effect was consistently stronger than that of sEV (Figure 2P; Figure S6). These results collectively demonstrate that Sm‐sEV attenuates inflammation and cellular senescence by potently inhibiting the NF‐κB signaling axis, in agreement with the transcriptomic data.

### 2.3 Construction and Characterization of αLA‐Gel for Sustained Sm‐sEV Delivery

**FIGURE 3:**
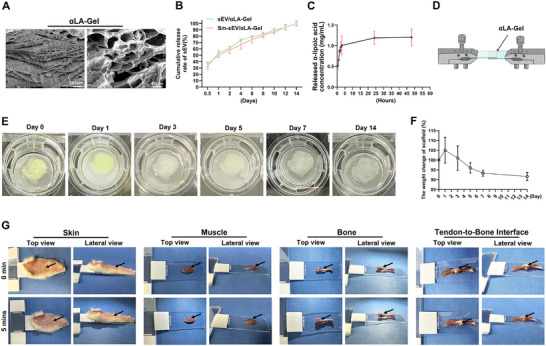
Characterization of Sm‐sEV/αLA‐Gel. (A) SEM image of the αLA‐Gel. (B) Release profile of Sm‐sEVs from the αLA‐Gel. (C) Release profile of αLA from the αLA‐Gel. (D) Schematic illustration of tensile adhesion testing. (E) Images of the Sm‐sEV/αLA‐Gel at different time points after incubation in SBF. (F) Degradation curve of the Sm‐sEV/αLA‐Gel. (G) Representative images demonstrating tissue adhesion of αLA‐Gel to skin, muscle, bone, and BTI. Black arrows indicate the location of αLA‐Gel. n = 3.

To improve the local retention and sustained release of Sm‐sEV, a tissue‐adhesive α‐lipoic acid‐based mucus‐like hydrogel was designed as a delivery platform. Its porous structure enabled efficient vesicle loading (Figure 3A) and sustained release for up to 14 days (Figure 3B), with α‐lipoic acid reaching the effective concentration of 1 mg/mL within 6 h (Figure 3C). The material exhibited a shear modulus of approximately 200 Pa (Figure 3D). At each time point, the mass loss of Sm‐sEV/αLA‐Gel in simulated body fluid (SBF) was measured, showing approximately 8.4% mass loss after 14 days, indicating stable degradation under these conditions (Figure 3E,F). Additionally, αLA‐Gel demonstrated stable adhesion to various tissue surfaces, including skin, muscle, bone, and bone‐tendon interfaces, while demonstrating excellent biocompatibility with BMSCs in vitro (Figure 3G; Figure S7).

### 2.4 Sm‐sEV/αLA‐Gel Mitigates Inflammation‐Aggravated BMSC Senescence and Restores Osteogenesis

**FIGURE 4:**
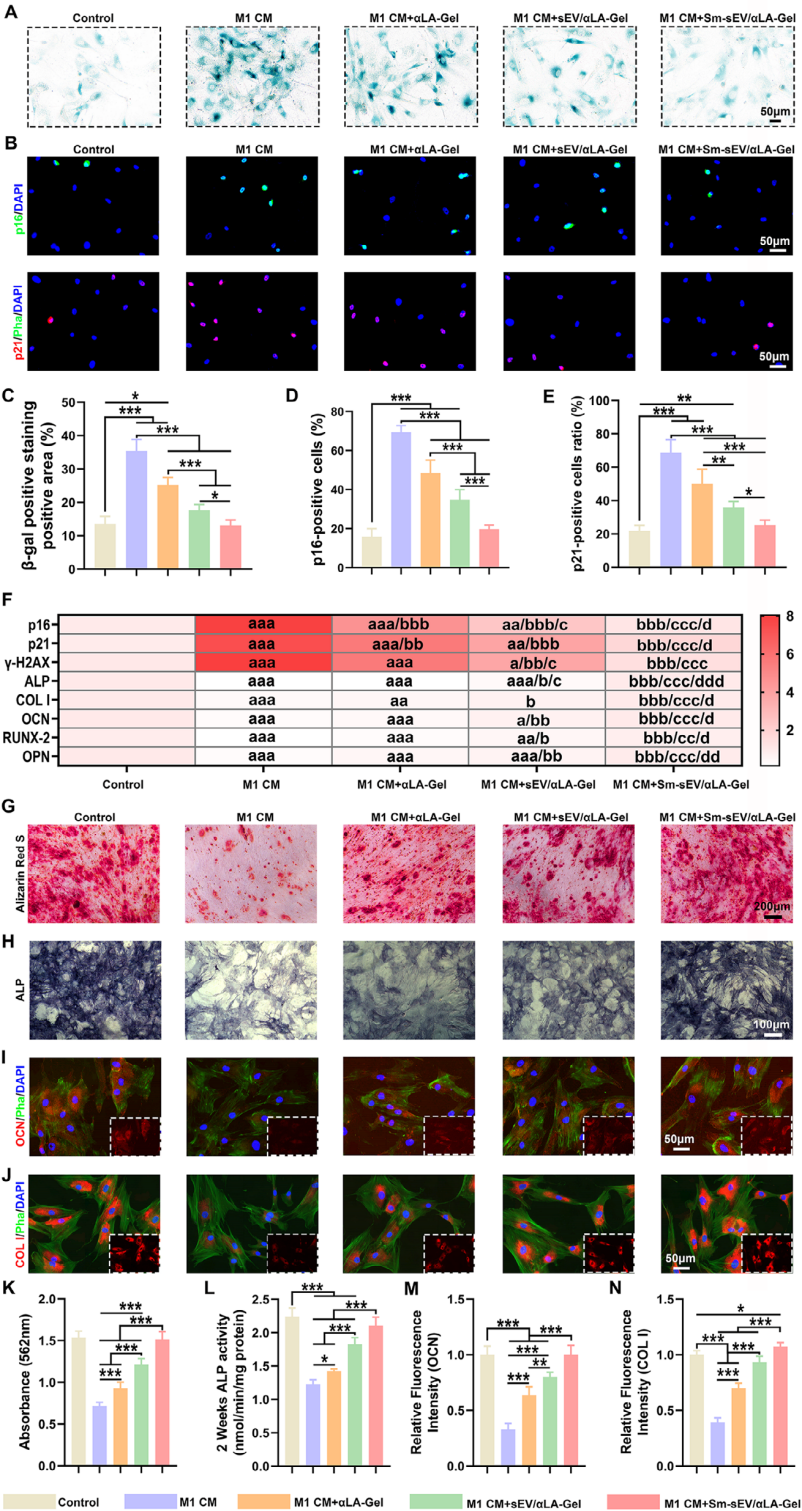
Sm‐sEV/αLA‐Gel alleviates inflammation‐exacerbated BMSC senescence and promotes osteogenesis. (A) SA‐β‐gal staining (B) Immunofluorescence staining for p16 and p21. (C–E) Quantitative analyses of SA‐β‐gal‐positive area and fluorescence intensity of p16 and p21. (F) Heatmap summarizing relative mRNA expression of senescence‐related genes (p16, p21, γ‐H2AX) and osteogenic markers (ALP, COL I, OCN, RUNX2, OPN) across treatment groups. (G,H) Alizarin Red S and ALP staining. (I,J) Immunofluorescence staining of osteogenic markers OCN and COL I. (K–N) Quantitative analyses of Alizarin Red absorbance, ALP activity, and fluorescence intensity of OCN and COL I. n = 3. ^*^ *p* < 0.05, ^**^ *p* < 0.01, ^***^ *p* < 0.001. a, aa, aaa indicate *p* < 0.05, *p* < 0.01, and *p* < 0.001 vs the Control group, respectively; b, bb, bbb indicate *p* < 0.05, *p* < 0.01, and *p* < 0.001 vs the M1 CM group, respectively; c, cc, ccc indicate p < 0.05, *p* < 0.01, and *p* < 0.001 vs the M1 CM + αLA‐Gel group; d, dd, ddd indicate *p* < 0.05, *p* < 0.01, and *p* < 0.001 vs the M1 CM +sEV/αLA‐Gel group, respectively.

To further evaluate the regulatory effects of Sm‐sEV on replicative senescent BMSCs under inflammatory stress, 10th‐generation BMSCs were exposed to M1 CM and treated with αLA‐Gel, sEV/αLA‐Gel, or Sm‐sEV/αLA‐Gel. SA‐β‐gal staining revealed a pronounced senescent phenotype in the M1 CM group, which was alleviated to varying degrees by all three interventions, with Sm‐sEV/αLA‐Gel showing the most significant reduction (Figure 4A,C). Consistently, immunofluorescence analysis showed strong upregulation of p16 and p21 in the M1 CM group, whereas Sm‐sEV/αLA‐Gel significantly suppressed their expression, outperforming both αLA‐Gel and sEV/αLA‐Gel (Figure 4B,D,E).

RT‐qPCR further demonstrated that M1 CM markedly increased the expression of senescence‐associated genes (p16, p21, γ‐H2AX) while suppressing osteogenesis‐related genes (ALP, collagen type I (COL I), RUNX2, OCN, OPN). Sm‐sEV/αLA‐Gel treatment effectively reversed these changes, resulting in the lowest expression levels of senescence markers and the highest expression of osteogenic markers, exceeding those of αLA‐Gel and sEV/αLA‐Gel (Figure 4F).

Functionally, Alizarin Red S and ALP staining showed that M1 CM markedly impaired mineralized nodule formation and ALP expression, whereas αLA‐Gel and Sm‐sEV/αLA‐Gel partially restored osteogenesis. Sm‐sEV/αLA‐Gel achieved the most pronounced recovery (Figure 4G,H), which was further supported by quantification of ‐Alizarin Red absorbance and ALP activity (Figure 4K,L). Immunofluorescence analysis revealed the highest OCN and COL I expression in the Sm‐sEV/αLA‐Gel group, with significantly stronger fluorescence intensity than αLA‐Gel and sEV/αLA‐Gel (Figure 4I,J,M,N).

Collectively, these results demonstrate that Sm‐sEV/αLA‐Gel more effectively attenuates BMSC senescence and restores osteogenic differentiation under inflammatory conditions compared with αLA‐Gel or Sm‐sEV/αLA‐Gel alone.

### 2.5 Sm‐sEV/αLA‐Gel Reverses Inflammation‐Aggravated Senescence in Replicative Aged BMSCs by Suppressing the cGAS‐STING‐NF‐κB Axis

**FIGURE 5:**
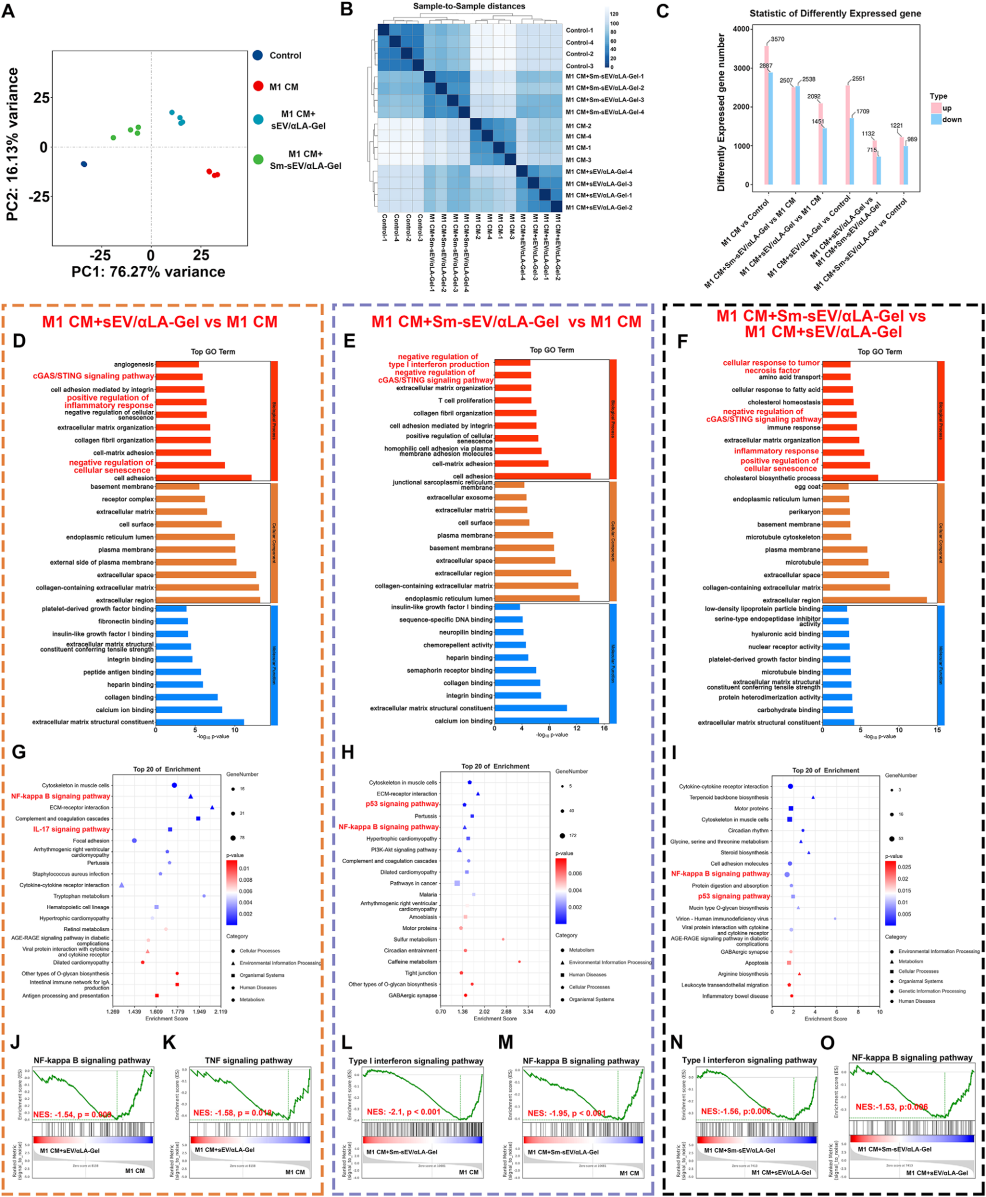
Transcriptomic and molecular characterization of Sm‐sEV/αLA‐Gel‐treated BMSCs subjected to inflammation‐exacerbated senescence. (A) PCA of gene expression profiles from Control, M1 CM, M1 CM+sEV/αLA‐Gel, and M1 CM+Sm‐sEV/αLA‐Gel groups (n = 4). (B) Sample‐to‐sample distance heatmap of RNA‐seq datasets. (C) Summary of differentially expressed genes between groups. (D–F) GO enrichment. (G–I) KEGG enrichment. (J–O) GSEA.

To elucidate the molecular mechanisms underlying the anti‐senescence effects of Sm‐sEV/αLA‐Gel, RNA‐seq was performed on replicative senescent BMSCs exposed to inflammatory stimulation (Control, M1 CM, M1 CM+sEV/αLA‐Gel, and M1 CM+Sm‐sEV/αLA‐Gel groups). Principal component analysis revealed distinct transcriptomic separation among the four groups (Figure 5A). Consistently, the distance heatmap and differential expression analyses showed that M1 CM markedly altered global gene expression, whereas Sm‐sEV/αLA‐Gel treatment largely restored the profile toward the control state (Figure 5B,C).

To assess transcriptomic changes induced by inflammatory stimulation, RNA‐seq comparing M1 CM and control BMSCs showed extensive differential gene expression. GO and KEGG analyses revealed enrichment in extracellular matrix organization, cyclic GMP‐AMP synthase (cGAS)‐stimulator of interferon genes (STING), NF‐κB, and p53 pathways, and cellular senescence. GSEA confirmed NF‐κB activation and suppression of DNA replication and cell cycle pathways, indicating a pro‐inflammatory and pro‐senescent transcriptional shift (Figure S8).

Volcano plot analysis showed widespread transcriptional alterations in both treatment groups relative to M1 CM, with Sm‐sEV/αLA‐Gel engaging a broader gene set (Figure S9), suggesting enhanced bioactivity following Sm modification. GO analysis showed that sEV/αLA‐Gel mainly enriched gene sets related to regulation of cGAS‐STING signaling, positive inflammatory regulation, and negative regulation of cellular senescence (Figure 5D). In contrast, Sm‐sEV/αLA‐Gel showed stronger enrichment in gene sets linked to negative regulation of type I interferon production and cGAS‐STING signaling, suggesting reduced activation of this inflammatory axis (Figure 5E,F). Consistently, both M1 CM + sEV/αLA‐Gel and M1 CM + Sm‐sEV/αLA‐Gel were enriched in processes including cellular response to tumor necrosis factor, negative regulation of cGAS‐STING signaling, and inflammatory response. KEGG pathway analysis showed that sEV/αLA‐Gel was enriched in NF‐κB and IL‐17 signaling pathways (Figure 5G), whereas Sm‐sEV/αLA‐Gel exhibited stronger enrichment in NF‐κB‐ and p53‐related pathways. Direct comparison further supported this trend, showing that Sm‐sEV had more prominent enrichment of NF‐κB and p53 pathways than sEV (Figure 5H,I). GSEA further validated these transcriptomic alterations. Compared with M1 CM, NF‐κB and TNF pathways were significantly downregulated in the sEV/αLA‐Gel group (Figure 5J,K). Sm‐sEV/αLA‐Gel treatment led to more pronounced downregulation of Type I interferon and NF‐κB signaling pathway (Figure 5L,M). Moreover, compared with sEV, Sm‐sEV showed stronger suppression of these inflammatory‐senescence signaling axes (Figure 5N,O).

**FIGURE 6:**
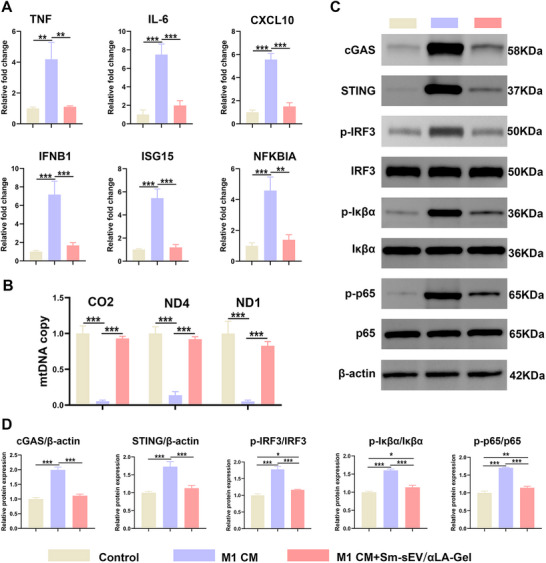
Analysis of cGAS‐STING‐NF‐κB signaling in BMSCs treated with M1 CM and Sm‐sEV/αLA‐Gel. (A) RT‐qPCR analysis of inflammatory and senescence‐associated genes. (B) Quantification of total mtDNA copy number (C) Western blot analysis of cGAS‐STING‐NF‐κB signaling‐related proteins and their phosphorylated forms. (D) Quantification of protein expression and phosphorylation ratios (n = 3). ^*^ *p* < 0.05, ^**^ *p* < 0.01, ^***^ *p* < 0.001.

qRT‐PCR confirmed transcriptional results, showing significant induction of TNF, IL‐6, CXCL10, IFNB1, ISG15, NFKBIA, IL‐1β, CCL2, and PTGS2 in the M1 CM group, which was markedly reversed by Sm‐sEV/αLA‐Gel treatment (Figure 6A; Figure S10). mtDNA quantification revealed a significant reduction in M1 CM‐treated cells, whereas Sm‐sEV/αLA‐Gel restored mtDNA copy number, suggesting mitigation of mitochondrial stress that is linked to mtDNA release and cGAS‐STING activation, based on total mtDNA measurement (Figure 6B).

At the protein level, western blot analysis further demonstrated that M1 CM upregulated cGAS and STING expression and enhanced phosphorylation of IRF3, IκBα, and p65, whereas Sm‐sEV/αLA‐Gel treatment markedly reversed these changes (Figure 6C‐D), confirming potent inhibition of the cGAS‐STING‐NF‐κB inflammatory axis. Furthermore, ELISA analysis of IL‐6 and CXCL10 in the conditioned medium revealed a significant reduction in both cytokines following Sm‐sEV/αLA‐Gel treatment, compared to M1 CM, further supporting the role of NF‐κB suppression in breaking the inflammation‐senescence cycle (Figure S11).

### 2.6 Sm‐sEV/αLA‐Gel Remodels the Inflammatory Microenvironment and Attenuates BMSC Senescence during Rotator Cuff Repair in Osteoporotic Rats

**FIGURE 7:**
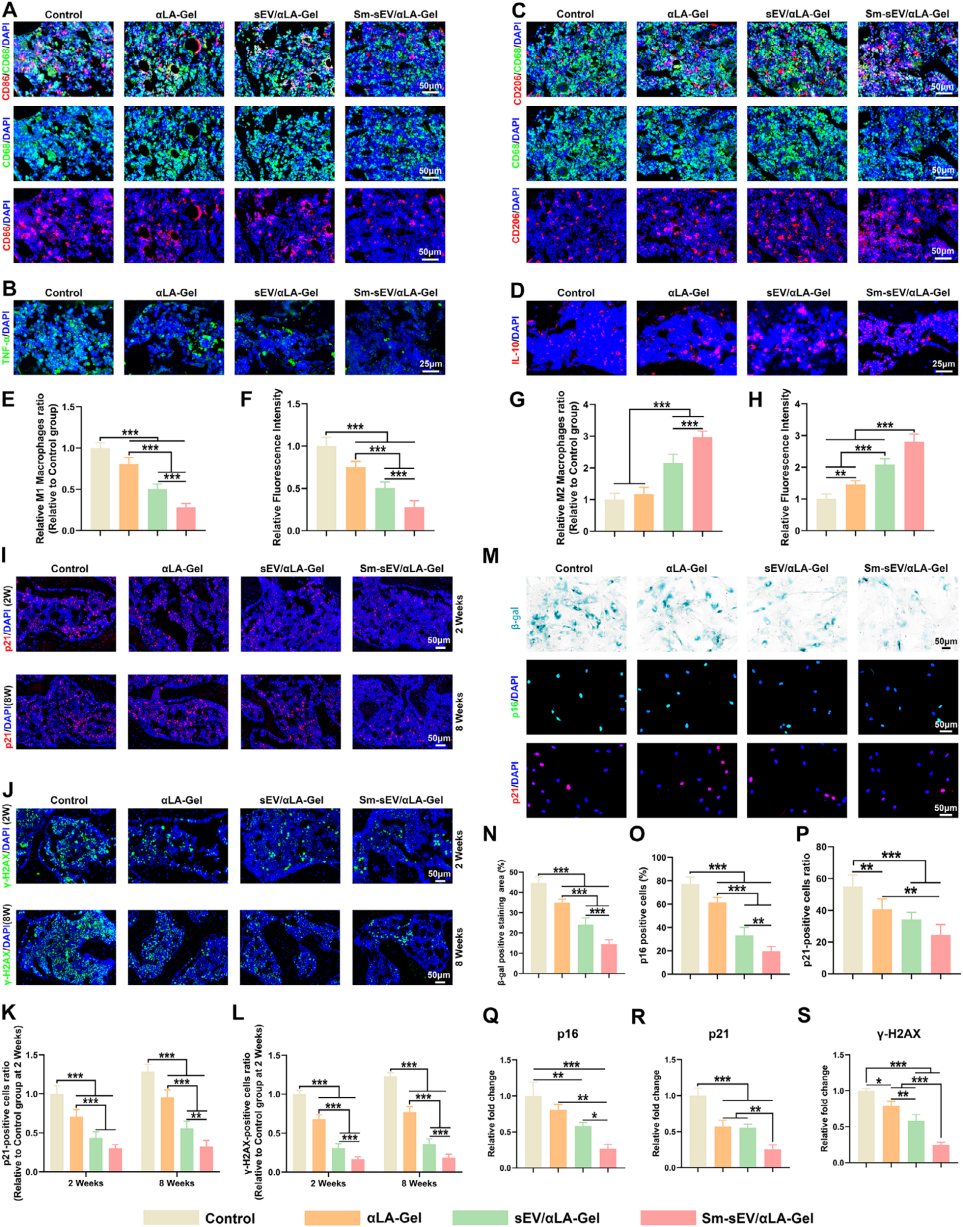
In vivo evaluation of Sm‐sEV/αLA‐Gel in modulating inflammation and cellular senescence during rotator cuff repair in osteoporotic rats. (A) Immunofluorescence staining of CD68/CD86 for M1 macrophages in rotator cuff tissue at 2 weeks post‐surgery. (B) Immunofluorescence staining of TNF‐α. (C) Immunofluorescence staining of CD68/CD206. (D) Immunofluorescence staining of IL‐10. (E–H) Quantitative analyses of M1 and M2 macrophage ratios and fluorescence intensities of TNF‐α and IL‐10. (I,J) Immunofluorescence staining for p21 and γ‐H2AX in the bone repair region at 2 and 8 weeks after surgery. (K,L) Quantitative analyses of p21‐ and γ‐H2AX‐positive cell ratios. (M–S) Analyses of BMSCs isolated from the humeral head 8 weeks after surgery. (M) SA‐β‐gal staining and immunofluorescence of p16 and p21. (N–P) Quantification of SA‐β‐gal‐positive area and p16^+^/p21^+^ cell ratios. (Q–S) RT‐qPCR analysis of senescence‐related genes (p16, p21, and γ‐H2AX). n = 6. ^*^ *p* < 0.05, ^**^ *p* < 0.01, ^***^ *p* < 0.001.

To investigate the in vivo therapeutic potential of Sm‐sEV/αLA‐Gel, a rotator cuff repair model was established in osteoporotic rats induced by OVX. During the repair process, αLA‐Gel, sEV/αLA‐Gel, or Sm‐sEV/αLA‐Gel was injected locally into the BTI. Immunofluorescence analysis of the injured region at 2 weeks post‐surgery revealed a clear shift in macrophage polarization and inflammatory cytokine expression. Double staining of CD68/CD86 showed abundant M1 macrophage infiltration in the control group, which was moderately reduced by αLA‐Gel and sEV/αLA‐Gel treatment but was most significantly decreased in the Sm‐sEV/αLA‐Gel group (Figure 7A,E). Consistently, TNF‐α expression was markedly lower in the Sm‐sEV/αLA‐Gel group than in other groups (Figure 7B,F). Conversely, CD68/CD206 double staining indicated a substantial increase in M2 macrophage polarization in the Sm‐sEV/αLA‐Gel group (Figure 7C,G). IL‐10 immunofluorescence further confirmed enhanced anti‐inflammatory cytokine expression in this group (Figure 7D,H).

Senescence‐associated markers were analyzed within the bone repair region at 2 and 8 weeks after surgery. Immunofluorescence and quantification of p21 and γ‐H2AX showed the highest levels in the control and αLA‐Gel groups, lower levels in the sEV/αLA‐Gel group, and the lowest in the Sm‐sEV/αLA‐Gel group. Although positive cell ratios increased over time in all groups, the rank order among treatment groups remained unchanged, with Sm‐sEV/αLA‐Gel exerting the strongest inhibitory effect. γ‐H2AX staining confirmed that Sm‐sEV/αLA‐Gel more effectively suppressed DNA damage signaling compared with other treatments (Figure 7I–L).

To further examine systemic effects, BMSCs were isolated from the humerus 8 weeks after surgery. SA‐β‐gal staining and p16/p21 immunofluorescence showed the highest senescence burden in the control group, limited improvement in the αLA‐Gel group, significant reduction in the sEV/αLA‐Gel group, and the lowest levels in the Sm‐sEV/αLA‐Gel group. Semi‐quantitative analysis confirmed significantly reduced SA‐β‐gal^+^ area and p16^+^/p21^+^ cell ratios in the Sm‐sEV/αLA‐Gel group. RT‐qPCR further showed minimal transcription of p16, p21, and γ‐H2AX in Sm‐sEV/αLA‐Gel‐treated BMSCs, followed by the sEV/αLA‐Gel group, with αLA‐Gel showing modest effects (Figure 7M–S).

### 2.7 Sm‐sEV/αLA‐Gel Promotes New Bone Formation and Collagen Matrix Remodeling to Accelerate Osteogenesis at the Rotator Cuff Repair Site

**FIGURE 8:**
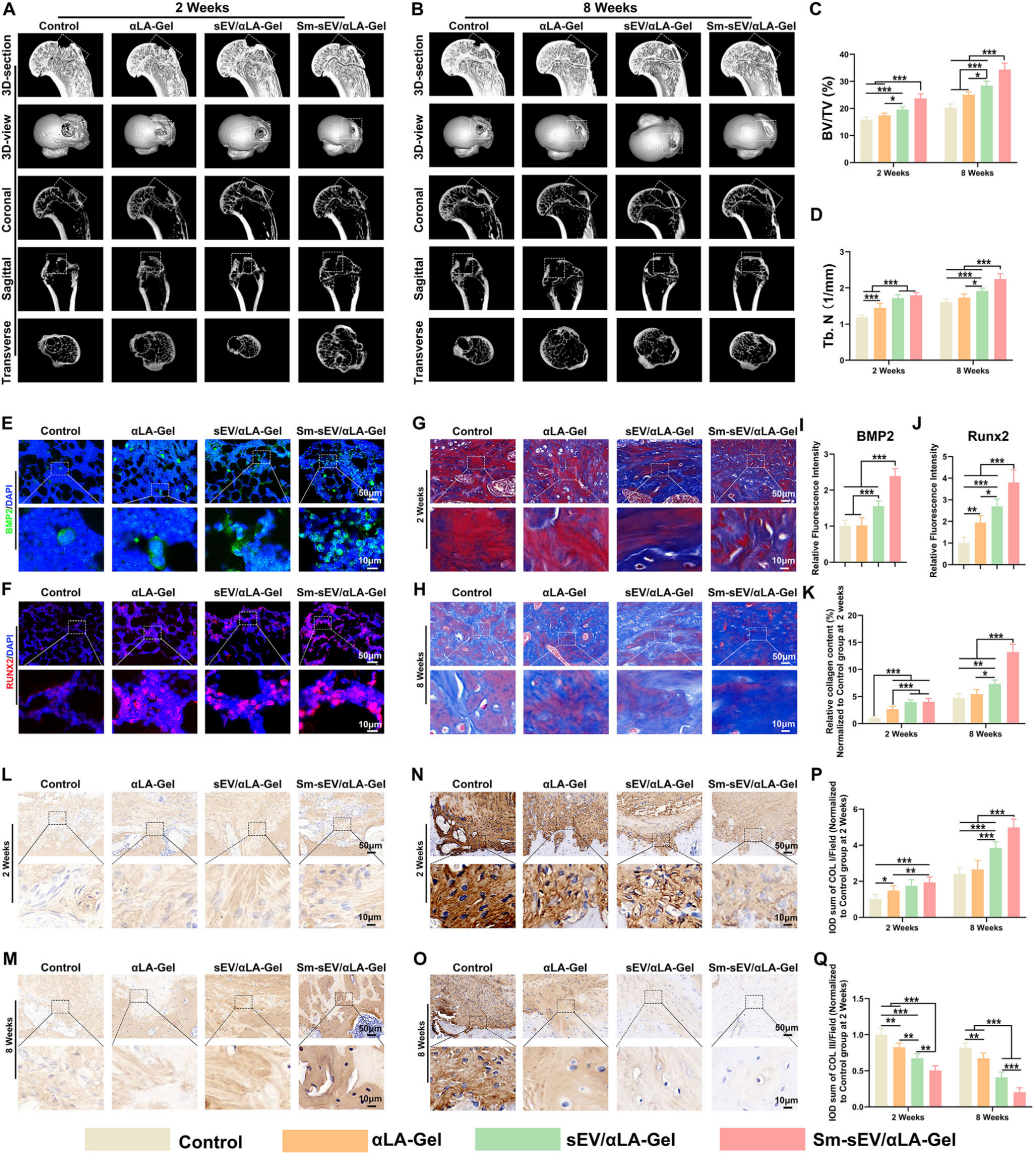
Evaluation of osteogenesis and collagen matrix remodeling at the rotator cuff repair site after Sm‐sEV/αLA‐Gel treatment in osteoporotic rats. (A,B) Representative micro‐CT images of the humeral head at 2 and 8 weeks post‐surgery. The white dashed boxes indicate the bone tunnel repair regions. (C,D) Quantitative analyses of BV/TV and Tb.N. (E,F) Immunofluorescence staining of BMP2 and RUNX2. (G–H) Masson's trichrome staining of the repair site at 2 and 8 weeks post‐surgery. (I,J) Quantitative analyses of BMP2 and RUNX2 fluorescence intensity. (K) Semi‐quantitative analysis of Masson's trichrome staining. (L,M) Immunohistochemical staining of COL I in the repair site. (N,O) Immunohistochemical staining of COL III in the same regions. (P,Q) Quantitative analyses of COL I and COL III expression ratios at 2 and 8 weeks post‐surgery. n = 6. ^*^ *p* < 0.05, ^**^ *p* < 0.01, ^***^ *p* < 0.001.

To assess the osteogenic effects of Sm‐sEV/αLA‐Gel in vivo, we evaluated bone regeneration in an osteoporotic rotator cuff repair model. Micro‐CT analysis at 2 and 8 weeks post‐surgery revealed significantly enhanced bone formation in the Sm‐sEV/αLA‐Gel group compared with the other groups, characterized by denser and more continuous new bone around the bone tunnel (Figure 8A,B). Quantitative analysis showed that BV/TV and Tb. N was consistently higher in the Sm‐sEV/αLA‐Gel group at both time points, indicating superior promotion of bone formation and trabecular reconstruction (Figure 8C,D). At 2 weeks, Tb. Th was higher in the sEV/αLA‐Gel and Sm‐sEV/αLA‐Gel groups than in the control, with the highest values in Sm‐sEV/αLA‐Gel. At 8 weeks, Tb. Th remained elevated in the sEV/αLA‐Gel group and was greatest in the Sm‐sEV/αLA‐Gel group. Tb. Sp showed the opposite trend, being higher in the control and αLA‐Gel groups than in Sm‐sEV/αLA‐Gel at 2 weeks, and consistently lowest in Sm‐sEV/αLA‐Gel at 8 weeks (Figure S12).

Immunofluorescence analysis of osteogenic markers further supported these findings. BMP2 and RUNX2 expression was highest in the Sm‐sEV/αLA‐Gel group, intermediate in sEV/αLA‐Gel, minimal in αLA‐Gel, and lowest in Control, indicating robust activation of osteogenesis‐related signaling pathways (Figure 8E,F,I,J). Masson's trichrome staining revealed markedly increased collagen deposition and osteoid formation in the Sm‐sEV/αLA‐Gel group at both 2 and 8 weeks, with semi‐quantitative analysis confirming enhanced early bone formation and tissue maturation (Figure 8G,H,K).

Immunohistochemistry showed clear differences in collagen remodeling among groups. At 2 weeks, COL I deposition was stronger in the Sm‐sEV/αLA‐Gel group than in the control and αLA‐Gel groups. By 8 weeks, staining intensified in all groups, with Sm‐sEV/αLA‐Gel showing the most evident collagen formation (Figure 8L,M,P). In contrast, Collagen type III (COL III) was highest in Control and αLA‐Gel, intermediate in sEV/αLA‐Gel, and lowest in Sm‐sEV/αLA‐Gel, with a time‐dependent decline (Figure 8N,O,Q).

### 2.8 Sm‐sEV/αLA‐Gel Further Promotes the Regeneration of Bone‐Attached Tissues, Including Fibrocartilage and Tendon

**FIGURE 9:**
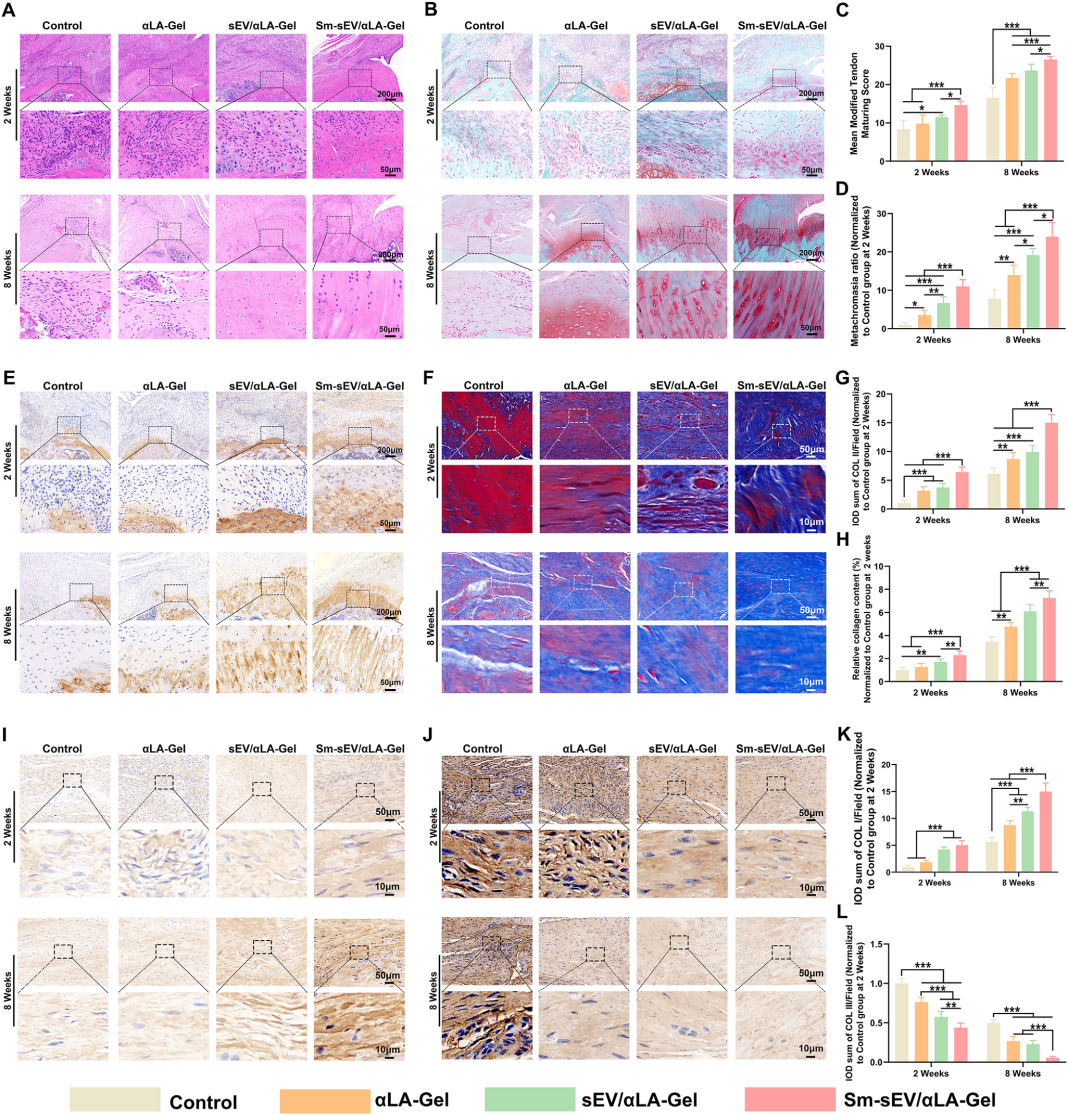
Histological and immunohistochemical evaluation of fibrocartilage regeneration and collagen remodeling in the BTI after Sm‐sEV/αLA‐Gel treatment. (A) H&E staining of the BTI at 2 and 8 weeks post‐surgery. (B) Safranin O/Fast Green staining of the BTI. (C,D) Quantitative analyses of histological scoring and Safranin O‐positive area. (E) Immunohistochemical staining of COL II in the BTI. (F) Masson's trichrome staining of the tendon region adjacent to the insertion site. (G,H) Quantitative analyses of COL II expression and Masson's trichrome staining intensity. (I,J) Immunohistochemical staining of COL I and COL III in the tendon region. (K,L) Quantitative analyses of COL I and COL III expression at 2 and 8 weeks post‐surgery. n = 6. ^*^ *p* < 0.05, ^**^ *p* < 0.01, ^***^ *p* < 0.001.

To examine whether Sm‐sEV/αLA‐Gel‐induced bone repair promotes the regeneration of adjacent fibrocartilage and tendon, these regions were analyzed in the osteoporotic rotator cuff model. Hematoxylin‐eosin (H&E) staining (Figure 9A), Safranin O/Fast Green staining (Figure 9B), and collagen type II (COL II) immunohistochemistry (Figure 9E) were performed to assess fibrocartilage formation at the bone attachment zone at 2 and 8 weeks post‐surgery. The Sm‐sEV/αLA‐Gel group showed a more continuous and organized fibrocartilage structure than the other groups at both time points. Correspondingly, histological scoring (Figure 9C), semi‐quantitative Safranin O analysis (Figure 9D), and COL II quantification (Figure 9G) were significantly higher in the Sm‐sEV/αLA‐Gel group, indicating enhanced fibrocartilaginous attachment associated with improved bone repair.

We further evaluated tendon remodeling near the fibrocartilage region using Masson's trichrome staining (Figure 9F) and immunohistochemistry for COL I (Figure 9I) and COL III (Figure 9J). The Sm‐sEV/αLA‐Gel group exhibited better‐aligned and denser collagen fibers, suggesting accelerated tendon matrix maturation. Quantitative analyses of Masson staining (Figure 9H), COL I (Figure 9K), and COL III (Figure 9L) confirm greater collagen deposition and remodeling in the Sm‐sEV/αLA‐Gel group compared with the others.

### 2.9 Sm‐sEV/αLA‐Gel Enhances Biomechanical Strength, Promotes Functional Recovery, and Exhibits Excellent In Vivo Biosafety

**FIGURE 10:**
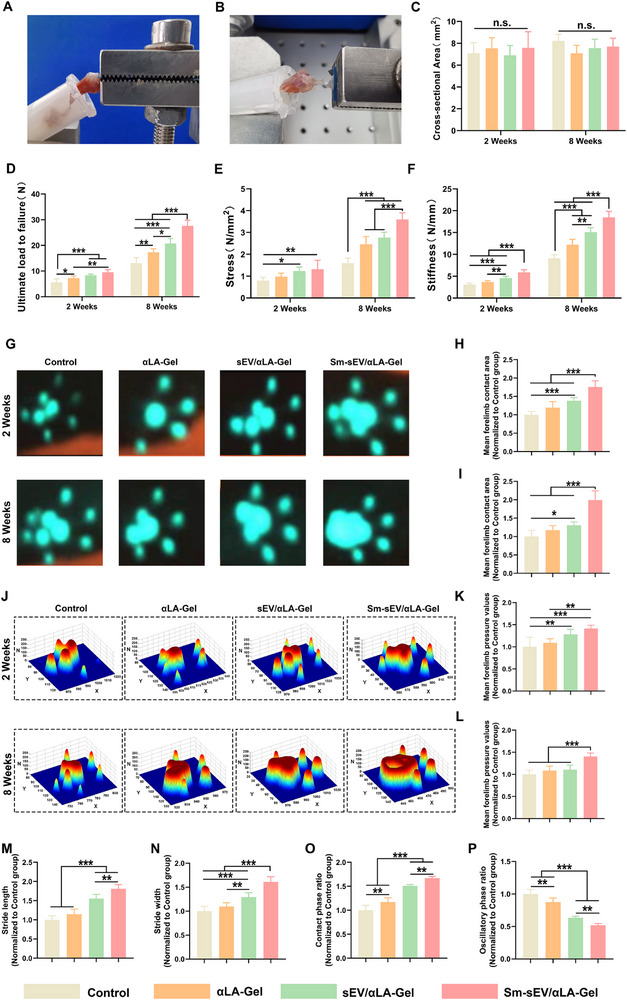
Biomechanical and functional evaluation of Sm‐sEV/αLA‐Gel‐mediated bone‐tendon healing. (A,B) Representative images of the biomechanical testing setup for rotator cuff repair specimens. (C) Quantitative analysis of the BTI cross‐sectional area. (D,F) Mechanical testing results showing ultimate load to failure, ultimate stress, and stiffness. (G) Representative forelimb gait pressure maps at 2 and 8 weeks. (H–I) Quantitative analyses of mean forelimb contact area normalized to the control group. (J) 3D gait pressure heatmaps showing dynamic pressure distribution. (K,L) Quantitative analyses of mean forelimb pressure values at 2 and 8 weeks. (M,N) Quantitative analyses of stride length and stride width were normalized to the control group at 8 weeks. (O,P) Quantitative analyses of contact phase ratio and oscillatory phase ratio at 8 weeks. n = 6. ^*^ *p* < 0.05, ^**^ *p* < 0.01, ^***^ *p* < 0.001.

To comprehensively evaluate the therapeutic performance of Sm‐sEV/αLA‐Gel, both biomechanical and functional assessments were performed in the osteoporotic rotator cuff repair model. Mechanical testing at 2 and 8 weeks post‐surgery revealed no significant differences in cross‐sectional area among the four groups (Figure 10A–C). However, the ultimate load to failure, ultimate stress, and stiffness exhibited a clear group‐dependent gradient. The Sm‐sEV/αLA‐Gel group achieved the highest biomechanical strength at both time points, followed by sEV/αLA‐Gel, whereas αLA‐Gel showed limited improvement, and the Control group was the lowest. All groups showed further enhancement at 8 weeks compared with 2 weeks, indicating progressive reinforcement of the BTI during healing (Figure 10D–F).

To assess postoperative functional recovery, gait pressure and kinematic analyses were conducted. Forelimb gait pressure maps revealed more uniform and continuous pressure distribution in the Sm‐sEV/αLA‐Gel group (Figure 10G). Quantitative analysis of contact area (Figure 10H,I) and pressure heatmaps (Figure 10J) demonstrated significantly greater load‐bearing capacity and higher mean pressure values (Figure 10K,L) compared with the other groups. Kinematic analysis further indicated that at 2 weeks post‐surgery, the Sm‐sEV/αLA‐Gel group already exhibited significantly longer stride length and greater stride width, while oscillatory and contact phase ratios remained comparable across groups (Figure S13). By 8 weeks, the Sm‐sEV/αLA‐Gel group showed the most pronounced functional improvement, with further increases in stride length (Figure 10M) and stride width (Figure 10N), along with stable contact (Figure 10O) and oscillatory phase ratios (Figure 10P). In addition, systemic biosafety was thoroughly assessed. Routine blood and biochemical analyses revealed no group‐related abnormalities in blood cell counts or hepatic and renal function markers (Figure S14). Furthermore, H&E staining of major organs, including the heart, liver, spleen, lung, and kidney (Figure S15), showed no evidence of structural abnormalities in any group.

## 3 Discussion

Regeneration of bone and the BTI in osteoporotic individuals remains one of the most formidable challenges in orthopedic and regenerative medicine [26, 27]. Successful repair at the BTI after rotator cuff injury requires the coordinated regeneration of bone, fibrocartilage, and tendon [28]. Among these processes, bone regeneration plays a decisive role by providing the structural foundation for interfacial healing and mechanical stability. However, in osteoporotic microenvironments, this regenerative cascade becomes profoundly impaired, characterized by persistent inflammation, reduced immune adaptability, and stem cell dysfunction [29, 30]. Our findings demonstrate that injury‐induced inflammation in an osteoporotic setting not only sustains a pro‐inflammatory niche but also accelerates BMSCs' senescence, leading to diminishing their osteogenic potential and ultimately impairing BTI regeneration. Thus, under osteoporotic conditions, inflammation impedes regeneration both by direct tissue injury and by aggravating stem cell senescence. Concurrently targeting chronic inflammation and enhancing stem cell resistance to inflammatory stress may therefore represent an effective strategy for age‐related musculoskeletal repair [31].

Persistent M1 macrophage infiltration after rotator cuff injury in osteoporotic rats was accompanied by increased BMSC senescence, suggesting that inflammation contributes to stem cell aging at the BTI. This appears to be an active inflammatory response rather than a passive consequence of aging, as supported by our in vitro finding that M1 CM directly induced BMSC senescence. These results are in line with emerging concepts that chronic inflammation accelerates cellular senescence and tissue degeneration, as observed in osteoporosis, osteoarthritis, and other age‐related musculoskeletal disorders [32, 33]. Compared with young tissues, aged tissues are more susceptible to inflammation‐exacerbated cellular senescence, creating a self‐reinforcing cycle that progressively deteriorates the regenerative niche [13, 34]. Although previous studies have mainly focused on regulating inflammation to promote tissue regeneration, they have largely overlooked the complex pathological milieu of osteoporosis under aging conditions, in which persistent inflammation renders stem cells particularly vulnerable and limits the efficacy of conventional anti‐inflammatory treatments. Our findings suggest that cellular senescence acts as a critical downstream barrier to regeneration, underscoring the need for therapeutic strategies that simultaneously target inflammation and senescence [35, 36].

To address these challenges, we applied a pharmacological preconditioning strategy using quercetin to reprogram donor cells, thereby generating sEV with enhanced senomorphic and immunomodulatory functions. Our data demonstrate that this strategy effectively interrupts the inflammation‐senescence feedback loop and restores a more favorable regenerative microenvironment under osteoporotic conditions. Consistent with our findings, quercetin‐preconditioned mesenchymal stem cell‐derived sEV have been reported to enhance anti‐inflammatory activity in intervertebral disc degeneration and osteoarthritis models [37, 38]. However, these investigations mainly focused on immunomodulation, whereas our results extend this concept by demonstrating that quercetin preconditioning also strengthens the senomorphic potential of sEV, directly mitigating inflammation‐induced stem cell aging. In a related study, Yang et al. developed quercetin‐loaded, CD86‐targeted exosomes that selectively cleared senescent microglia and mitigated neuroinflammation after stroke [19]. Unlike such post‐loading strategies, our preconditioning approach modulates sEV bioactivity during biogenesis, allowing the coordinated incorporation of bioactive molecules while maintaining vesicle integrity. This method avoids the drawbacks of post‐loading processes, such as inconsistent encapsulation efficiency and potential structural disruption, while preserving the natural composition and functionality of sEV [39, 40]. Together, these results demonstrate that pharmacological preconditioning offers a simple but effective strategy to generate functionally enhanced sEV, providing a biologically integrated platform for targeting inflammation‐ and senescence‐associated degeneration. Notably, proteomic analysis revealed enrichment of GDF11 in Sm‐sEV. GDF11 is a transforming growth factor‐β superfamily ligand that has been implicated in aging‐related regulation and tissue remodeling, although its reported effects in bone appear context‐dependent across models and conditions [41]. We therefore speculate that enrichment of GDF11 in Sm‐sEV may contribute, as part of the overall cargo, by providing localized cues that support the resilience of BMSCs under inflammatory stress. The specific contribution of GDF11 warrants further investigation.

Mechanistically, transcriptomic and molecular analyses identified suppression of the NF‐κB signaling pathway as a key downstream effect of Sm‐sEV treatment. Sm‐sEV markedly reduced phosphorylation of IκBα and p65, as well as pro‐inflammatory cytokines including TNF‐α, IL‐6, and CXCL10, indicating its ability to dampen inflammatory signaling and mitigate inflammation‐induced BMSC senescence. These results highlight that Sm‐sEV primarily acts through downstream inhibition of NF‐κB, a central node linking inflammation and cellular senescence in aged tissues [42].

Aberrant activation of the cGAS‐STING pathway is increasingly recognized as a key pathogenic mechanism in degenerative musculoskeletal disorders, including osteoarthritis, intervertebral disc degeneration, and osteoporosis [43, 44, 45]. In aging tissues, DNA damage and mitochondrial dysfunction lead to cytosolic mtDNA leakage, which chronically activates cGAS‐STING signaling and drives inflammation‐induced stem cell senescence and regenerative decline [46]. Modulation of this pathway using STING inhibitors or mitochondrial stabilizers has been shown to alleviate senescence‐associated inflammation and improve tissue repair in degenerative contexts [47, 48]. In our study, Sm‐sEV treatment attenuated NF‐κB‐related inflammatory and senescence‐associated signaling in BMSCs, and its combination with αLA‐Gel further suppressed cGAS–STING activation, indicating cooperative regulation across multiple levels of the pathway. This mechanism‐driven rejuvenation contrasts with conventional regenerative strategies, which often show limited efficacy in aged or osteoporotic tissues due to persistent inflammatory and senescent barriers [49, 50].

Rather than directly stimulating repair, our platform alleviates upstream inflammatory and senescence‐associated constraints, thereby restoring a microenvironment supportive of progenitor cell function [51]. Reversal of BMSC senescence is central to this process, as senescent progenitors exhibit impaired osteogenesis, reduced matrix deposition, and disrupted paracrine signaling essential for fibrocartilage maturation and tendon anchorage [52]. Enhanced bone regeneration likely provides the primary structural and mechanical basis for subsequent fibrocartilage maturation and tendon anchorage, while local immunomodulation further facilitates matrix remodeling across the interface [53]. Together, these effects promote osteotendinous integration and support improved enthesis regeneration, highlighting the translational promise of this bioactive system. Importantly, both components offer practical advantages. α‐Lipoic acid possesses a well‐established safety profile, and ADSC‐derived sEV were selected as a practical source because adipose tissue is abundant and relatively less invasive to harvest, and has been reported to provide higher EV yield and stable culture performance, supporting scalable production for therapeutic use [54, 55]. The injectable, minimally invasive format further supports its application to other degenerative musculoskeletal disorders, including ligament, spinal, and osteoporotic repair contexts.

Nevertheless, several limitations should be acknowledged. The OVX‐induced osteoporotic model was selected instead of naturally aged animals because it reproduces key aging‐related features, including estrogen‐deficiency‐driven bone loss, chronic inflammation, and stem cell dysfunction, while offering superior experimental control and reproducibility. In contrast, naturally aged models require prolonged induction and exhibit high individual variability, which complicates mechanistic interpretation. Although transcriptomic and biochemical analyses strongly implicate the cGAS‐STING‐NF‐κB pathway, further studies using genetic or pharmacological perturbation will be necessary to establish causality. In addition, the long‐term safety, biodistribution, and potential off‐target effects of Sm‐sEV/αLA‐Gel require more extensive evaluation before clinical translation. From a translational perspective, scalable Good Manufacturing Practice‐compliant manufacturing with standardized quality control for both sEVs and the hydrogel, together with long‐term safety and immunogenicity assessment and an sEV‐biomaterial combination‐product regulatory strategy, will be essential. Moreover, aging is a multifactorial process, and how different aging phenotypes and comorbidities influence the efficacy of this therapy remains to be explored.

## 4 Conclusion

Chronic inflammation and inflammation‐exacerbated BMSC senescence jointly create a hostile regenerative microenvironment that severely limits bone and BTI repair under osteoporotic conditions. By integrating senomorphic sEV generated through quercetin preconditioning with a tissue‐adhesive αLA‐Gel, we developed a dual‐functional immuno‐rejuvenating platform that simultaneously suppresses inflammatory signaling and alleviates inflammation‐exacerbated BMSC senescence. Mechanistically, the composite mitigates this heightened senescence response in part through inhibition of the cGAS‐STING‐NF‐κB pathway. In vivo, the Sm‐sEV/αLA‐Gel system reconstructs the regenerative microenvironment, restores osteogenic capacity, promotes fibrocartilage regeneration and tendon repair, and enhances biomechanical integrity at the BTI. With its biocompatibility and translational potential, this platform offers a promising therapeutic approach for musculoskeletal regeneration in aging and osteoporosis. More broadly, these findings underscore the inflammation‐senescence interplay as a pivotal mechanistic and therapeutic axis in regenerative medicine.

## 5 Experimental Section

### 5.1 Animal Models and Surgical Procedures

All animal procedures were approved by the Institutional Animal Care and Use Committee of Shanghai Sixth People's Hospital (DWLL2025‐1047). Female Sprague‐Dawley rats (12 weeks old, 220–250 g) underwent bilateral OVX to induce systemic osteoporosis. Animals were wild‐type (non‐genetically modified) and were purchased from Shanghai Jihui Laboratory Animal Breeding Co., Ltd. Rats were housed under standard Specific Pathogen‐Free conditions with free access to food and water. All surgical procedures were performed under inhalational anesthesia using 2% isoflurane, and postoperative analgesia was administered via subcutaneous injection of buprenorphine (0.05 mg/kg) to minimize discomfort. Twelve weeks after OVX, rotator cuff surgery was performed on both shoulders of each rat. Animals were randomly assigned to different experimental groups and sacrificed at predetermined time points. To ensure data integrity, tissues used for primary BMSC isolation were not used for histological or biomechanical assessments. A total of 99 rats were used; 3 animals were excluded due to perioperative complications or accidental death. At designated time points, animals were humanely euthanized by CO_2_ inhalation followed by cervical dislocation, and tissues were collected immediately for further analysis.

#### 5.1.1 Rotator Cuff Injury Model (for Inflammation‐Senescence Analysis)

A rotator cuff tear model was established by detaching the supraspinatus tendon from the greater tuberosity without repair. Rats were sacrificed at day 0, 3, 7, 14, 28, and 56 (n = 6 rats per time point, 12 shoulders per time point). Samples were allocated to micro‐CT, histology/immunofluorescence, or BMSC isolation as mutually exclusive endpoints.

#### 5.1.2 Rotator Cuff Repair Model (for Therapeutic Intervention)

After tendon detachment, a 4 mm bone tunnel was drilled at the footprint, followed by tendon reinsertion and fixation using a modified transosseous technique. Rats were randomly assigned to five groups: Control, αLA‐Gel, sEV/αLA‐Gel, Sm‐sEV/αLA‐Gel, and Sham (n = 6 rats per group per time point). The Control group underwent OVX and rotator cuff repair with PBS injection at the BTI, while the Sham group received no OVX and no rotator cuff surgery. For the αLA‐Gel, sEV/αLA‐Gel, and Sm‐sEV/αLA‐Gel groups, 30 µL of the respective formulation containing sEV or Sm‐sEV at a concentration of 5 × 10^9 mL^−1^ was injected directly into the BTI region. Animals were evaluated at 2 and 8 weeks (n = 6 rats per group per time point; 12 shoulders per group per time point). Gait analysis was performed before sacrifice.

### 5.2 Cell Sources and Culture Conditions

The human monocytic cell line THP‐1 (RRID: CVCL_0006; serial no. SCSP‐567) was purchased from the Cell Bank of the Chinese Academy of Sciences (Shanghai, China). Human ADSCs and BMSCs were obtained from Zhong Qiao Xin Zhou Biotechnology Co., Ltd. (Shanghai, China). THP‐1 cells were cultured in RPMI‐1640 medium supplemented with 10% fetal bovine serum (FBS) and 1% penicillin‐streptomycin at 37°C in a humidified atmosphere containing 5% CO_2_. To induce macrophage differentiation, THP‐1 cells were stimulated with 100 ng mL^−^^1^ phorbol 12‐myristate 13‐acetate for 24 h, followed by washing with PBS and culturing in fresh medium for another 24 h to obtain M0 macrophages. M1 polarization was induced by treatment with 100 ng mL^−^^1^ lipopolysaccharide (LPS) for 24 h, while M2 polarization was achieved by stimulation with 20 ng mL^−^^1^ IL‐4 for 24 h. Human ADSCs and BMSCs were maintained in α‐MEM containing 10% FBS and 1% penicillin‐streptomycin under standard culture conditions. Human ADSCs at passages 3–5 were used for subsequent in vitro experiments, whereas passage 10 BMSCs were used as a replicative senescence model.

### 5.3 Micro‐CT Imaging and Bone Morphometric Analysis

Micro‐computed tomography was used to evaluate both pathological bone degeneration following rotator cuff injury and new bone formation during repair. Scans were performed using a SkyScan 1176 system (Bruker, Germany) at 70 kV and 200 µA with an isotropic voxel size of 18 µm. Image reconstruction was carried out with the manufacturer's software, and 3D analysis was performed using CTAn (version 1.13, Bruker). The region of interest (ROI) was defined as the humeral head region encompassing the supraspinatus tendon insertion and the bone tunnel area. Morphometric parameters included BV/TV, Tb. Th, Tb. N, and Tb. Sp.

### 5.4 Histology, Immunohistochemistry, and Immunofluorescence

Tissues were fixed in 4% paraformaldehyde, decalcified, paraffin‐embedded, and sectioned at 5 µm thickness. Histology: H&E staining was used for general morphology, Safranin O/Fast Green for fibrocartilage evaluation, and Masson's trichrome for collagen deposition and organization. BTI healing was evaluated using a modified tendon maturing score system as previously described (Table S3) [56]. For collagen detection, sections underwent antigen retrieval, 3% H_2_O_2_ quenching, and 5% BSA blocking, followed by incubation with primary antibodies against COL I, COL III, and COL II overnight at 4°C. HRP‐conjugated secondary antibodies and DAB were used for signal development, followed by hematoxylin counterstaining. Images were acquired under identical microscope settings. Quantitative analysis was performed using Image‐Pro Plus (IPP). For each section, the ROI was manually delineated, and the integrated optical density (IOD) and positive staining area were measured. The mean IOD value (IOD/area) was calculated to represent relative expression levels. For inflammatory, senescence, and osteogenic markers, sections were stained with primary antibodies (CD68, CD86, CD206, TNF‐α, IL‐10, p21, γ‐H2AX, BMP2, RUNX2, and OCN), followed by fluorescent secondary antibodies and DAPI counterstaining. Confocal microscopy was used for imaging. All primary antibodies used in IHC and IF experiments are listed in Table S1.

### 5.5 Isolation and Culture of Rat BMSCs

Rat BMSCs were isolated from the humeral heads of OVX rats. Bone marrow was flushed with α‐MEM (10% FBS, 1% penicillin–streptomycin), filtered through a 70 µm strainer, and plated. After 48 h, non‐adherent cells were removed, and adherent BMSCs were maintained in complete α‐MEM at 37°C with 5% CO_2_. Passage 1 cells were used for in vitro assays requiring preservation of in vivo cellular states.

### 5.6 Sm‐sEV Preparation, Characterization, and Proteomic Analysis

Small extracellular vesicles were isolated from human ADSCs by differential ultracentrifugation. Briefly, ADSCs were cultured to 80% confluence. After PBS washing, ADSCs were maintained in exosome‐depleted FBS‐containing medium. For Sm‐sEV, cells were treated with 20 µm quercetin for 48 h, then switched to fresh exosome‐depleted medium for conditioned‐medium collection over the following 48 h. Culture supernatants were sequentially centrifuged at 300 × g for 10 min, 2 000 × g for 20 min, and 10 000 × g for 30 min to remove cells and debris, followed by ultracentrifugation at 100 000 × g for 70 min. The sEV pellet was washed with PBS and ultracentrifuged again at 100 000 × g for 70 min, resuspended in PBS, and stored at −80°C. The morphology of sEV and Sm‐sEV was assessed by TEM, particle size distribution by NTA, and Western blotting was performed for exosome markers (CD9, CD81, and HSP70) and the negative marker calnexin. For proteomic profiling, label‐free quantitative proteomics was performed by OE Biotech (Shanghai, China). Briefly, proteins were extracted from sEV and Sm‐sEV, digested with trypsin, and analyzed using LC‐MS/MS. Raw data were searched against the UniProt human database. Differentially expressed proteins were defined by fold change > 1.5 and p‐value < 0.05, and functional enrichment was performed using GO, KEGG, and GSEA analysis to identify inflammation‐ and senescence‐related signatures.

### 5.7 Macrophage Polarization and Conditioned Medium, and Assays on Replicative‐Senescent BMSCs

THP‐1 cells were differentiated into M0 macrophages with PMA (100 ng mL^−^^1^) for 24 h, then cultured in PMA‐free medium for 24 h. M0 cells were polarized to M1 with LPS (100 ng mL^−^^1^) or to M2 with IL‐4 (20 ng mL^−^^1^) for 24 h. Conditioned media were collected, centrifuged (300 × g, then 2000 × g), and filtered through 0.22 µm filters. For polarization assays, macrophages were treated with sEV or Sm‐sEV (5 × 10^9 mL^−^^1^) for 24 h. RT‐qPCR was performed for M1 (IL‐1β, TNF‐α, IL‐6) and M2 (Arg‐1, IL‐1ra, IL‐10) genes. IF staining of iNOS and CD206 was used to visualize polarization. For senescence assays, P10 human BMSCs were cultured in M1 CM (1:1 with growth medium) for 48 h to exacerbate senescence, followed by treatment with PBS, sEV, or Sm‐sEV (5 × 10^9 mL^−^^1^) for an additional 48 h. Senescence was assessed by SA‐β‐gal staining, IF and RT‐qPCR for p16, p21, γ‐H2AX, and inflammatory mediators (IL‐6, TNF, IL‐1β, CCL2, PTGS2, NFKBIA). Osteogenesis was induced with osteogenic medium containing dexamethasone, β‐glycerophosphate, and ascorbic acid. Alizarin Red S staining and ALP staining were performed at the indicated time points to assess mineralization. Quantitative analysis of ARS absorbance and ALP activity was conducted using a microplate reader. All primary antibodies used are listed in Table S1, and RT‐qPCR primer sequences are provided in Table S2.

### 5.8 RNA Sequencing and Bioinformatics

Total RNA was extracted from BMSCs using TRIzol reagent, and RNA quality was confirmed by agarose gel electrophoresis and Bioanalyzer assessment. Library construction and sequencing were performed by OE Biotech (Shanghai, China). Sequencing reads were processed, aligned, and analyzed using the Oebiotech cloud platform (https://cloud.oebiotech.cn/task/). A fold change > 2 and a *p*‐value < 0.05 were considered significant. Differentially expressed genes were subjected to GO, KEGG, and GSEA.

### 5.9 αLA‐Gel Platform Preparation and Characterization

The αLA‐Gel was synthesized as follows: 0.17 g of sterile GelMA was dissolved in 5 mL of sterilized H_2_O at 55 degrees Celsius. After GelMA was fully dissolved, 0.27 g of Tris (hydroxymethyl)aminomethane was added before 0.7 g of α‐lipoic acid was dissolved. Then, the mixture was heated at 55 degrees Celsius for 4 h to obtain the αLA‐Gel. 0.5 mL of αLA‐Gel was injected into the mold before it was frozen at −80°C for 24 h. After the αLA‐Gel was fully frozen, it was placed into a vacuum freeze‐dryer and freeze‐dried for 48 h. Then, the freeze‐drying αLA‐Gel was observed by SEM scanning (Hitachi, SU8220). A syringe was used to extract 0.5 mL of α‐lipoic acid gel, which was then evenly spread between two plastic sheets. The covered area and thickness were recorded. The sheets were vertically fixed at both ends of a universal force gauge, and a slow, constant tensile force was applied while recording the force‐displacement relationship. The stretching shear modulus of the α‐lipoic acid gel was calculated from the recorded data. The 1 gram of Sm‐sEV/αLA‐Gel was incubated in 4 mL of simulated body fluid (SBF) at 37°C. The samples were collected at the following time points: Day 0, 1, 3, 5, 7, and 14. At each time point, the mass loss was measured. The tissue adhesion of αLA‐Gel was assessed on skin, muscle, bone, and bone‐tendon surfaces. sEV loading efficiency and release kinetics were evaluated over 14 days. Biocompatibility was tested by co‐culturing with BMSCs.

### 5.10 Assays on Replicative‐Senescent BMSCs Treated with αLA‐Gel, sEV/αLA‐Gel, and Sm‐sEV/αLA‐Gel

Replicative‐senescent human BMSCs were seeded in 6‐well plates for RT‐qPCR analysis and in 24‐well plates for SA‐β‐gal staining, immunofluorescence staining, and osteogenic differentiation assays. Cells were exposed to M1 CM (1:1 with growth medium) for 48 h to induce inflammatory stress, followed by treatment with αLA‐Gel, sEV/αLA‐Gel, or Sm‐sEV/αLA‐Gel for an additional 48 h, while PBS‐treated cells served as controls. Senescence was evaluated by SA‐β‐gal staining, and the percentage of positive cells was calculated from five random fields per sample. Immunofluorescence staining for p16 and p21 was performed to assess protein expression levels. Total RNA was isolated and analyzed by RT‐qPCR to evaluate senescence‐associated genes (p16, p21, γ‐H2AX) and osteogenesis‐related genes (ALP, COL I, RUNX2, OCN, OPN), with GAPDH as the internal control, and data were analyzed using the 2^–ΔΔCt^ method. Osteogenic differentiation was induced with osteogenic medium containing dexamethasone, β‐glycerophosphate, and ascorbic acid, followed by ALP staining and ARS staining. Quantitative analysis of ALP activity and ARS absorbance was performed using a microplate reader at 562 nm. Immunofluorescence staining for OCN and COL I was conducted, followed by Alexa Fluor‐conjugated secondary antibodies and DAPI counterstaining. Images were captured using confocal microscopy, and integrated fluorescence intensity was quantified using ImageJ software. The primary antibodies used in this study are summarized in Table S1, and the RT‐qPCR primer sequences are listed in Table S2.

### 5.11 qRT‐PCR, Mitochondrial DNA Quantification, Western Blotting, and ELISA

To validate RNA‐seq results and assess inflammatory signaling, total RNA was extracted from BMSCs using Trizol‐up, and reverse transcription was performed using a cDNA synthesis kit. qRT‐PCR was conducted with SYBR Green qPCR Master Mix (EZBioscience) to analyze the expression of TNF, IL‐6, CXCL10, IFNB1, ISG15, NFKBIA, IL‐1β, CCL2, and PTGS2, with GAPDH as the internal control. Relative gene expression was calculated using the 2^–ΔΔCt^ method. Mitochondrial DNA copy number was determined by qPCR using total DNA extracted with a genomic DNA extraction kit (Tiangen, China). Results were expressed as relative mtDNA copy number. For protein analysis, cells were lysed in RIPA buffer containing protease and phosphatase inhibitors. Equal amounts of protein were separated by SDS‐PAGE and transferred onto PVDF membranes. After blocking with 5% non‐fat milk, membranes were incubated overnight at 4°C with primary antibodies against cGAS, STING, p‐IRF3, IRF3, p‐IκBα, IκBα, p‐p65, p65, and β‐actin, followed by HRP‐conjugated secondary antibodies. Protein bands were visualized using ECL and quantified with ImageJ software. The primary antibodies used in this study are summarized in Table S1, and the RT‐qPCR primer sequences are listed in Table S2. IL‐6 and CXCL10 levels in the conditioned medium of BMSCs treated with M1‐conditioned medium and Sm‐sEV/αLA‐Gel were measured using Thermo Fisher Scientific ELISA kits, following the manufacturer's instructions.

### 5.12 Gait and Biomechanical Testing

Gait analysis was performed using the VisuGait gait analysis system (Shanghai Xinruan Information Technology Co., Ltd., Shanghai, China). During testing, rats traversed a transparent walkway from one end to the other, and their footprints and plantar pressure profiles were recorded using a high‐speed camera positioned beneath the platform. Key gait parameters, including mean forelimb pressure, mean contact area, stride length, stride width, and the ratios of contact and oscillatory phases, were automatically derived using the system's analysis software. All values were normalized to those of the Control group to minimize inter‐individual variability and enable direct comparisons across groups.

Biomechanical testing was performed on freshly harvested supraspinatus‐humeral complexes at 2 and 8 weeks post‐surgery. After carefully removing soft tissues, the tendon attachment area was measured using digital calipers. The specimens were mounted on a mechanical testing machine (HSS‐DX1000, Jinan Heng Rui Jin Testing Machine Co., Ltd., China). A preload of 0.1 N was applied, followed by uniaxial tensile loading at a displacement rate of 10 mm/min until failure. The ultimate load, ultimate stress, and stiffness were recorded. Stiffness was determined from the linear portion of the load‐load‐displacement curve, and stress was calculated by dividing the ultimate load by the cross‐sectional area at the tendon insertion site.

### 5.13 Biosafety Evaluation

In vitro: BMSCs were seeded in 96‐well plates at a density of 1 × 10^3^ cells per well and allowed to adhere overnight. Cells were then incubated with αLA‐Gel, sEV/αLA‐Gel, or Sm‐sEV/αLA‐Gel. Cell viability was evaluated using the CCK‐8 assay at 0 and 3 days. At each time point, 10 µL of CCK‐8 reagent was added to each well and incubated for 2 h. The absorbance was measured at 450 nm using a microplate reader. For the Live/Dead staining assay, cells were stained with calcein AM and propidium iodide (PI), and images were acquired using a DMi8 microscope (Leica, Germany).

In vivo: Hematological and serum biochemical analyses were performed at 8 weeks post‐surgery to assess systemic biosafety. Blood samples were collected from the abdominal aorta under anesthesia. Hematological indicators included white blood cells (WBC), red blood cells (RBC), lymphocytes, monocytes, granulocytes, hemoglobin, and platelets. Biochemical parameters included alanine aminotransferase (ALT), aspartate aminotransferase (AST), blood urea nitrogen (BUN), creatinine, and uric acid. Major organs (heart, liver, spleen, lung, and kidney) were harvested, fixed in 4% paraformaldehyde, embedded in paraffin, sectioned, and subjected to H&E staining.

### 5.14 Statistical Analysis

All experiments were performed at least in triplicate. Data are presented as mean ± SD. Statistical significance was determined using an unpaired Student's t‐test for two groups or one‐way ANOVA with Tukey's post hoc test for multiple groups. A value of *p* < 0.05 was considered statistically significant. Analyses were performed using GraphPad Prism 9.0.

## Conflicts of Interest

The authors declare no conflicts of interest.

## Supporting information




**Supporting File**: advs74847‐sup‐0001‐SuppMat.pdf.

## Data Availability

The data that support the findings of this study are available on request from the corresponding author. The data are not publicly available due to privacy or ethical restrictions.
