# Supplementary material for: Senomorphic Small Extracellular Vesicles Delivered by a Tissue‐Adhesive α‐Lipoic‐Acid Hydrogel Enable Immuno‐Rejuvenation for Bone‐Tendon Interface Regeneration
Source: Adv Sci (Weinh). 2026 Apr 7;13(30):e24366. doi: 10.1002/advs.202524366 (PMC13248854; doi:10.1002/advs.202524366)
Supplement: Supplementary file 1 — Supporting File: advs74847‐sup‐0001‐SuppMat.pdf. [file ADVS-13-e24366-s001.pdf]

Supporting Information for

Senomorphic Small Extracellular Vesicles Delivered by a Tissue-Adhesive α-Lipoic-Acid Hydrogel Enable Immuno-Rejuvenation for Bone-Tendon Interface Regeneration

Lingzhi Kong, Wei Song, Wencai Liu, Hui Xu, Yuhao Yu, Xinyue Yang, Haiyan Li, Yanlun Zhu*, Yaohua He**

1. Figures

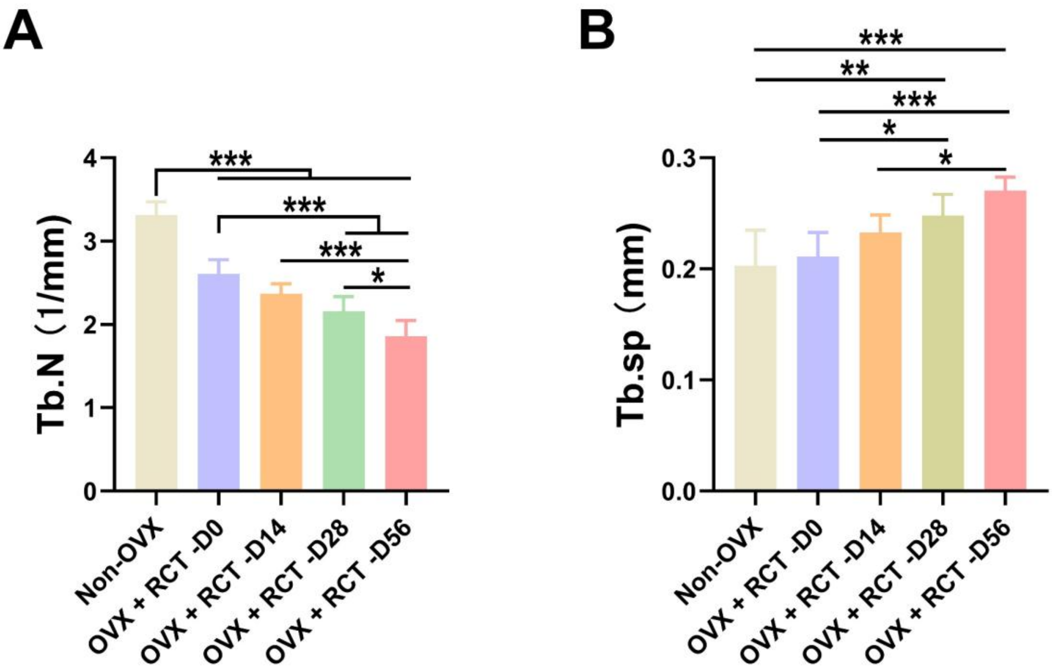

Figure S1. Quantitative assessment of trabecular bone microstructure in the osteoporotic RCT model. (A) Tb.N and (B) Tb.Sp in the humeral head of Non-OVX and OVX rats at different time points after RCT induction (D0, D14, D28, D56). *p < 0.05, **p < 0.01, ***p < 0.001.

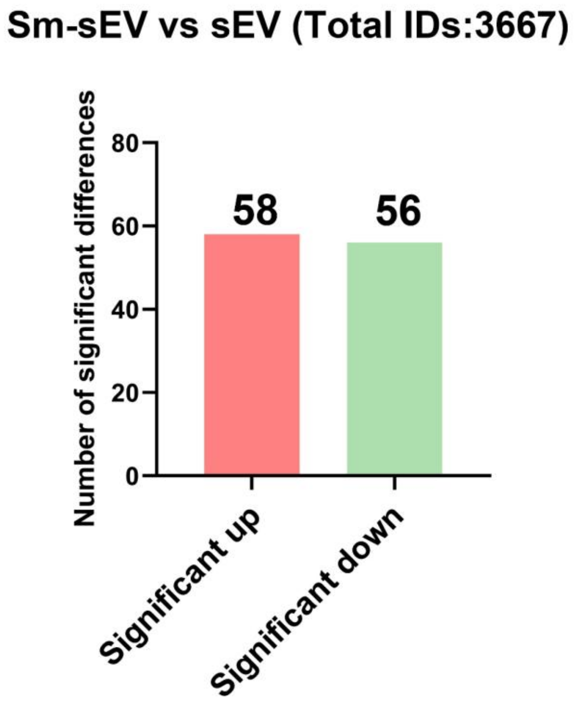

Figure S2. Quantitative analysis of differentially expressed proteins between Sm-sEV and sEV.

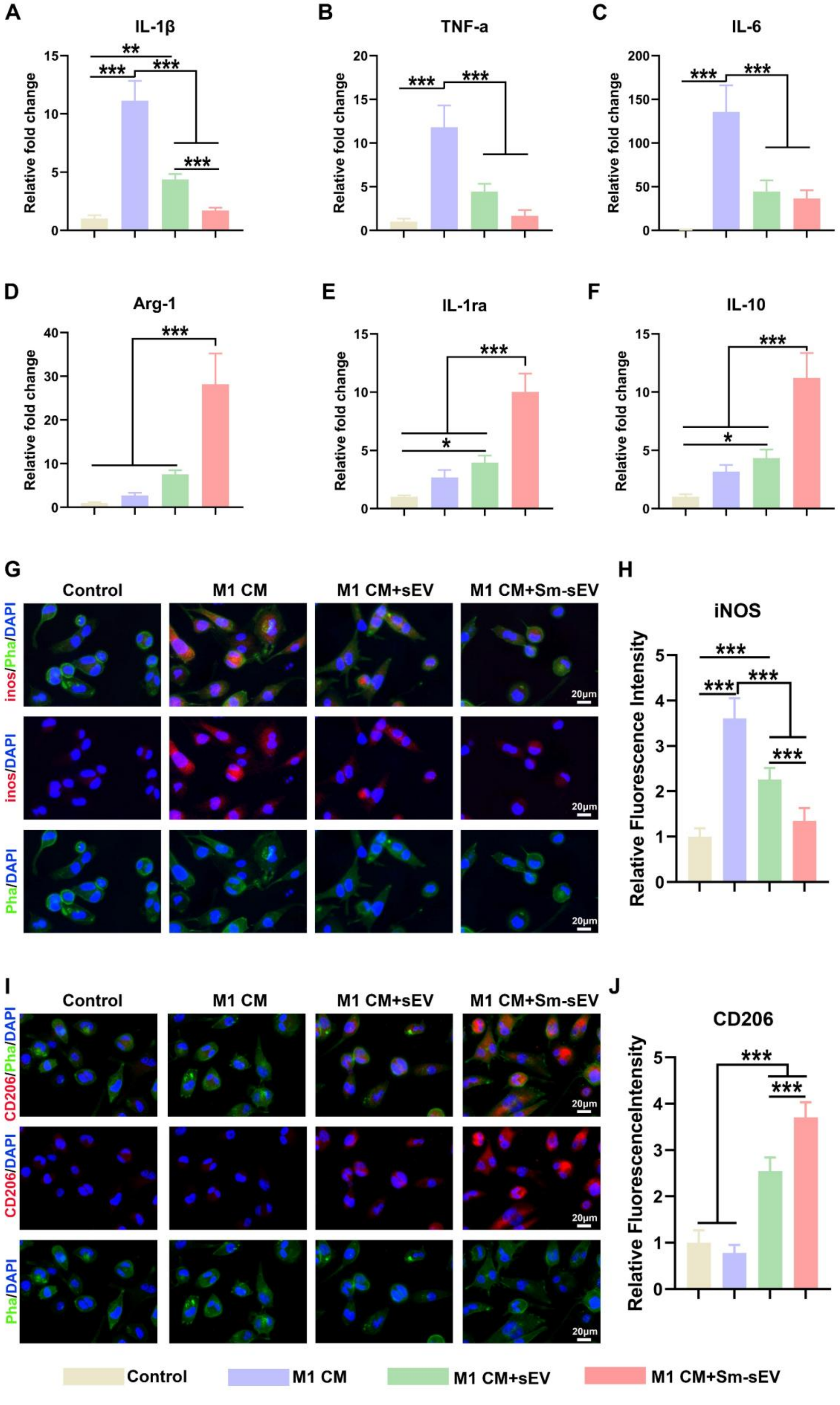
A
IL-1β
B
TNF-a
C
IL-6
D
Arg-1
E
IL-1ra
F
IL-10
Relative fold change
G
Control
M1 CM
M1 CM+sEV
M1 CM+Sm-sEV
inos/Pha/DAPI
inos/DAPI
Pha/DAPI
20μm
H
iNOS
Relative Fluorescence Intensity
I
CD206/Pha/DAPI
CD206/DAPI
J
CD206
Relative FluorescenceIntensity
Control
M1 CM
M1 CM+sEV
M1 CM+Sm-sEV

Figure S3. Sm-sEV enhances macrophage polarization toward the M2 phenotype and suppresses M1-type inflammatory responses. (A–C) RT-qPCR analysis of pro-inflammatory cytokines IL-1β, TNF-α, and IL-6 expression levels in macrophages treated with M1 CM, M1 CM with sEV, or M1 CM with Sm-sEV. (D–F) Expression of anti-inflammatory and M2-related markers Arg-1, IL-1ra, and IL-10. (G) Representative immunofluorescence images showing iNOS (red) and phalloidin (green) staining in macrophages across groups. (H) Quantification of iNOS fluorescence intensity. (I) Representative immunofluorescence images showing CD206 (red), iNOS (red), and phalloidin (green) expression. (J) Quantification of CD206 fluorescence intensity. Nuclei were counterstained with DAPI (blue). $*p < 0.05$, $**p < 0.01$, $***p < 0.001$.

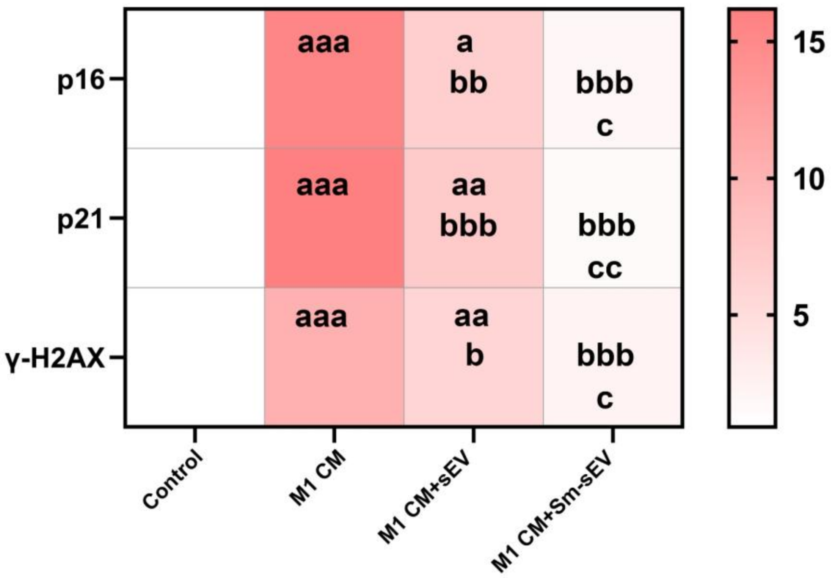

Figure S4. Sm-sEV alleviates inflammatory stress-exacerbated senescence in BMSCs at the transcriptional level. Heatmap showing the relative mRNA expression levels of senescence- and DNA damage-related markers p16, p21, and γ-H2AX in BMSCs treated with control medium, M1 CM, M1 CM supplemented with sEV, or M1 CM supplemented with Sm-sEV. a, aa, aaa indicate $p < 0.05$, $p < 0.01$, and $p < 0.001$ versus the Control group, respectively; b, bb, bbb indicate $p < 0.05$, $p < 0.01$, and $p < 0.001$ versus the M1 CM group, respectively; c, cc, ccc indicate $p < 0.05$, $p < 0.01$, and $p < 0.001$ versus the M1 CM + sEV group, respectively.

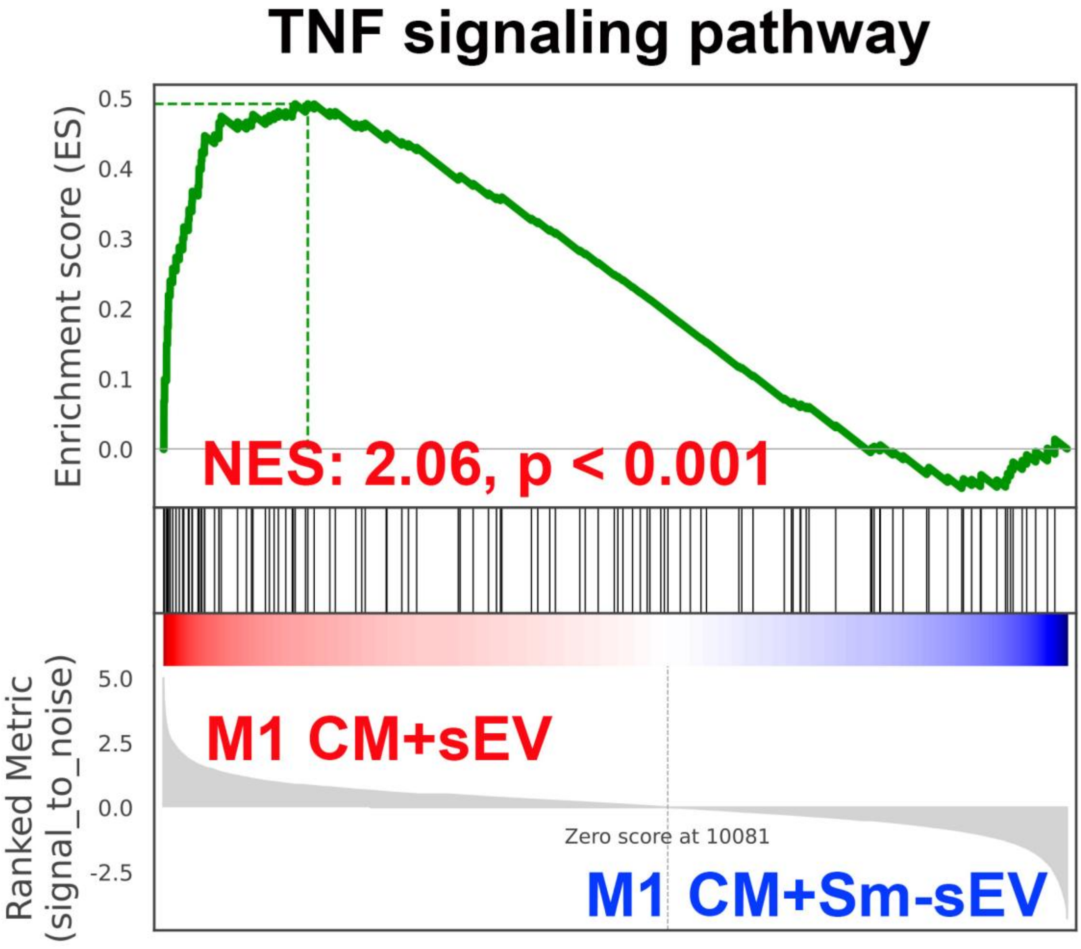

Figure S5. GSEA reveals suppression of TNF signaling in Sm-sEV-treated cells compared with sEV.

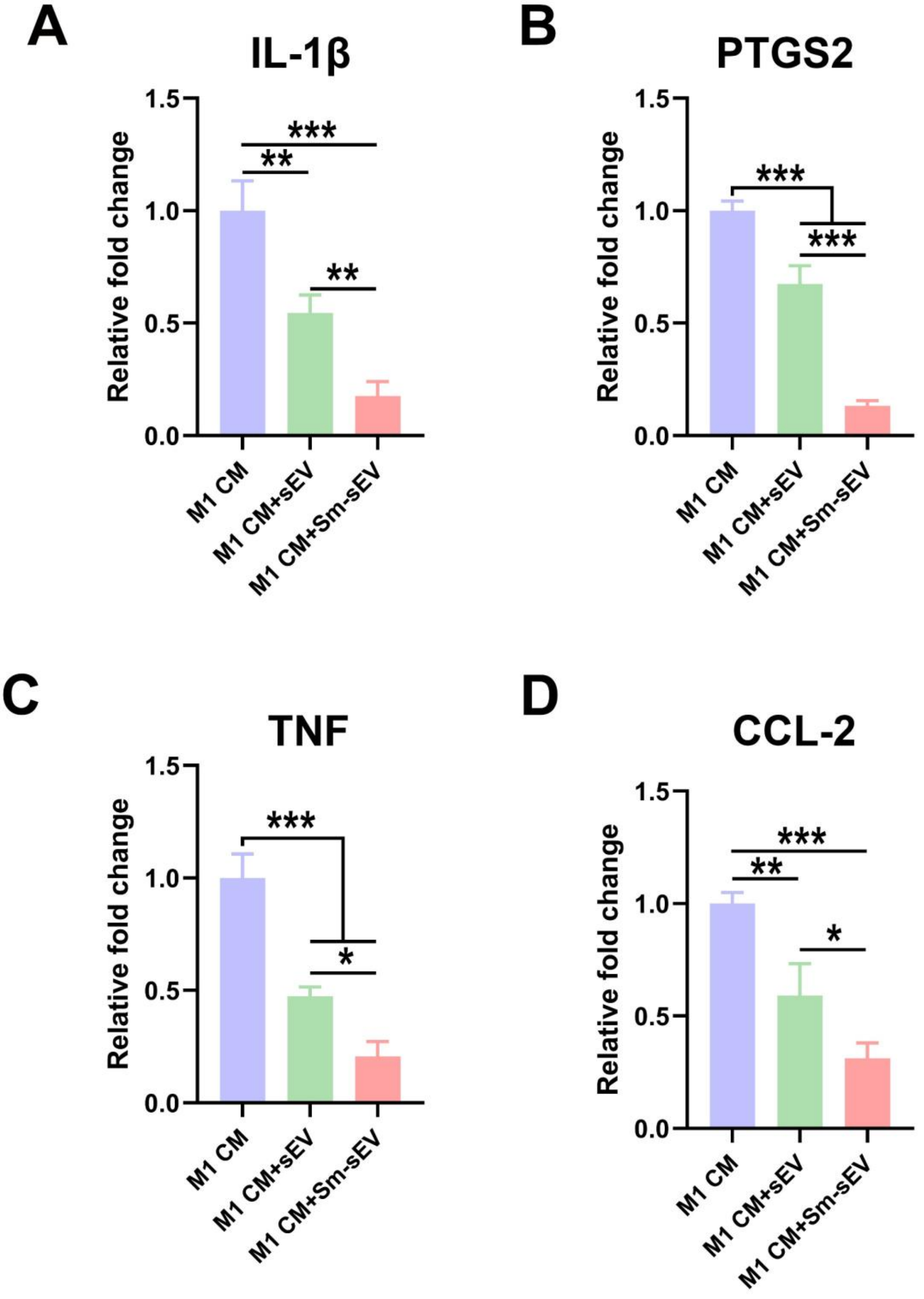

Figure S6. Sm-sEV exhibits enhanced inhibitory effects on NF-κB downstream pro-inflammatory gene expression compared with sEV. RT-qPCR analysis showing the relative mRNA expression levels of (A) *IL-1β*, (B) *PTGS2*, (C) *TNF,* and (D) *CCL-2* in BMSCs treated with M1 CM, M1 CM supplemented with sEV, or M1 CM supplemented with Sm-sEV. *p < 0.05, **p < 0.01, ***p < 0.001.

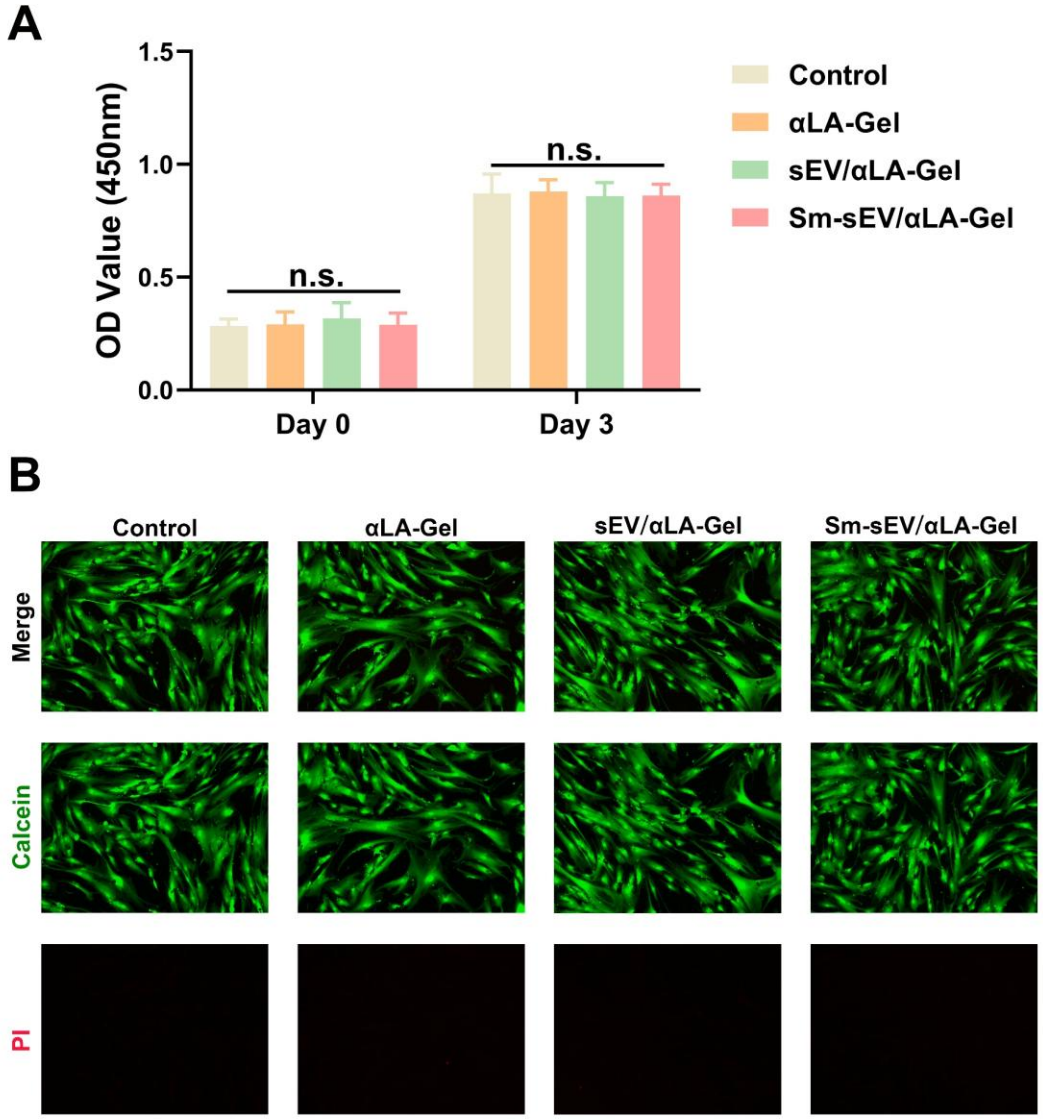

Figure S7. Biocompatibility evaluation of Sm-sEV/αLA-Gel with BMSCs. (A) CCK-8 assay showing the OD values of BMSCs cultured with different treatments (Control, αLA-Gel, sEV/αLA-Gel, and Sm-sEV/αLA-Gel) on Days 0 and 3. (B) Live-dead staining of BMSCs treated with different conditions on Day 3. PI; propidium iodide. n.s., not significant.

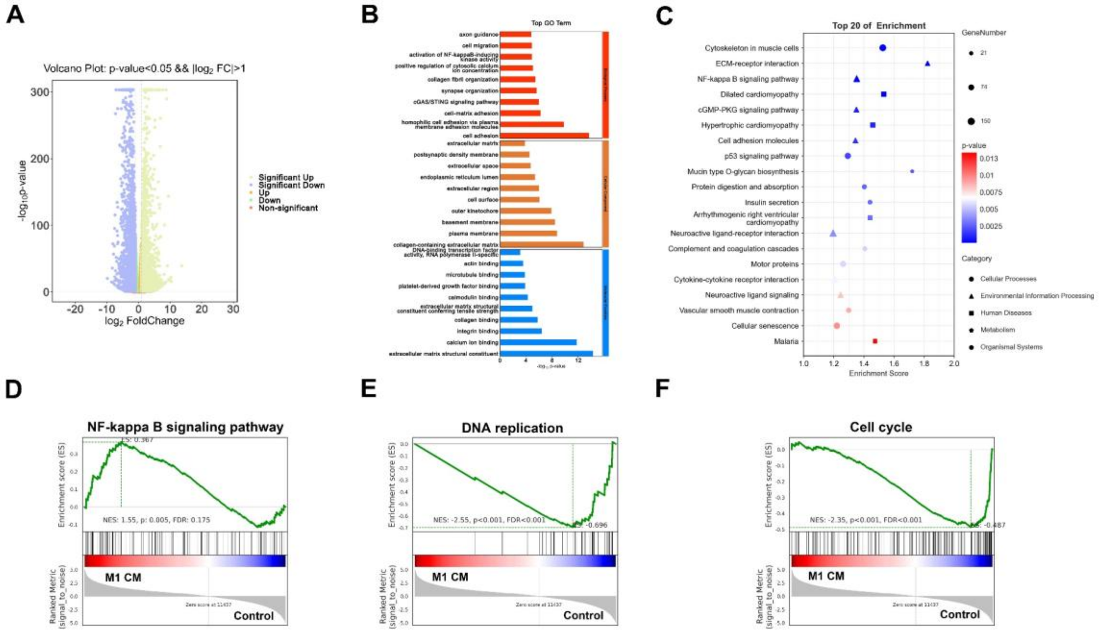

Figure S8. Transcriptomic analysis of BMSCs under inflammatory stimulation. (A) Volcano plot showing differentially expressed genes between M1 CM-treated and control BMSCs. (B) GO enrichment analysis of significantly altered genes. (C) KEGG pathway enrichment analysis. (D–F) GSEA plots of representative pathways, including NF-κB signaling (D), DNA replication (E), and cell cycle (F).

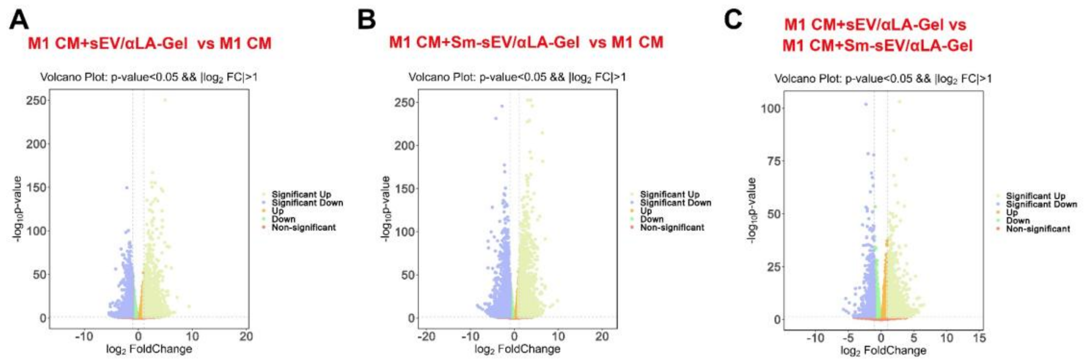

Figure S9. Volcano plot analysis of differentially expressed genes among treatment groups. (A) Volcano plot showing differentially expressed genes between M1 CM and M1 CM + sEV/αLA-Gel. (B) Volcano plot showing differentially expressed genes between M1 CM and M1 CM + Sm-sEV/αLA-Gel. (C) Volcano plot showing differentially expressed genes between M1 CM + sEV/αLA-Gel and M1 CM + Sm-sEV/αLA-Gel.

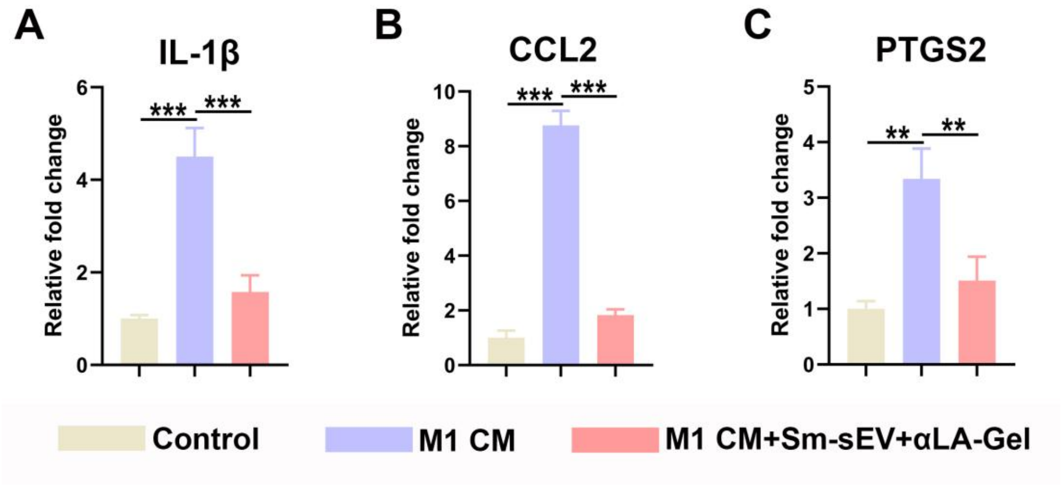

Figure S10. Quantitative RT-PCR analysis of inflammatory gene expression in BMSCs. (A–C) Relative mRNA expression levels of IL-1β (A), CCL2 (B), and PTGS2 (C) in Control, M1 CM, and M1 CM + Sm-sEV/αLA-Gel groups. $*p < 0.05$, $**p < 0.01$, $***p < 0.001$.

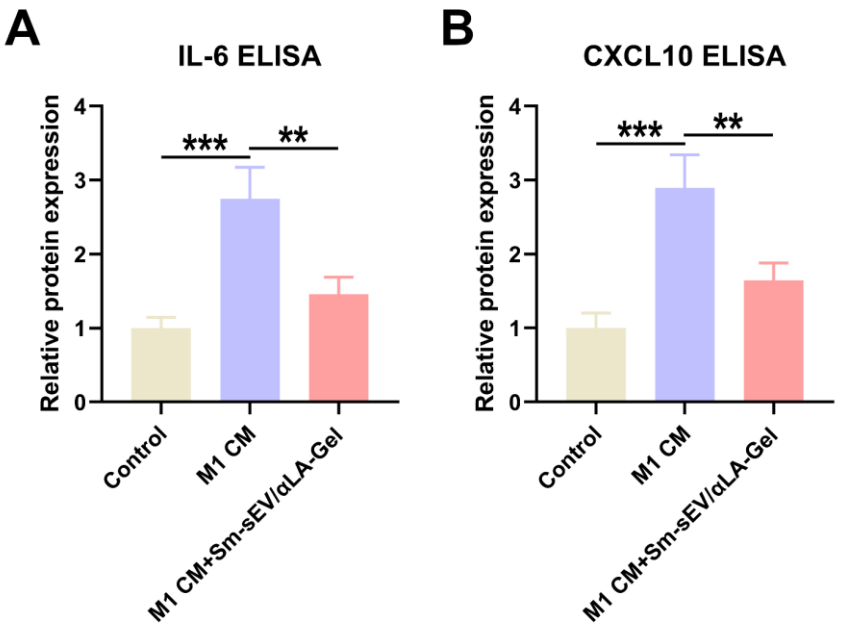

Figure S11. Quantification of SASP factors (IL-6 and CXCL10) in the conditioned medium of BMSCs treated with M1-conditioned medium and Sm-sEV/αLA-Gel. (A) IL-6 expression levels. (B) CXCL10 expression levels. $^{**}p < 0.01$, $^{***}p < 0.001$.

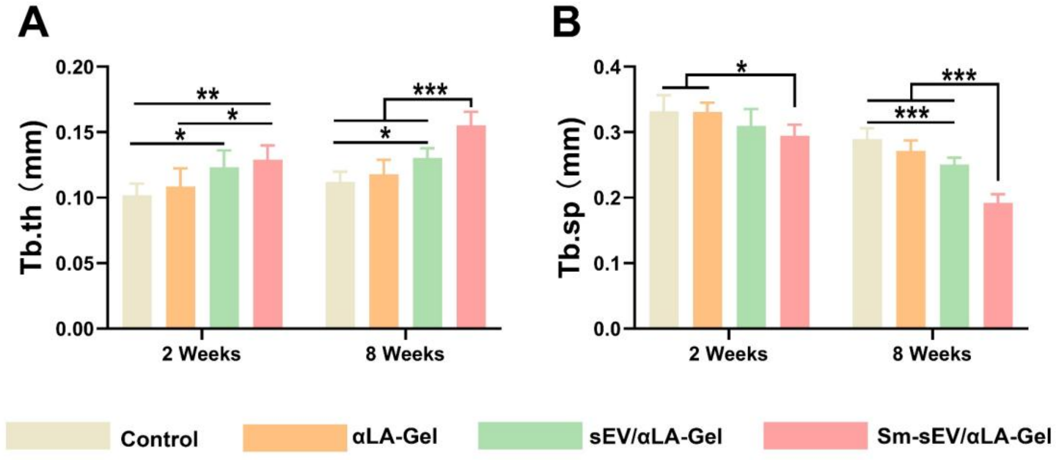

Figure S12. Quantitative analysis of trabecular bone parameters following different treatments. (A) Tb.th and (B) Tb.sp of bone zone at 2 and 8 weeks post-treatment in Control, αLA-Gel, sEV/αLA-Gel, and Sm-sEV/αLA-Gel groups. *$p < 0.05$, **$p < 0.01$, ***$p < 0.001$.

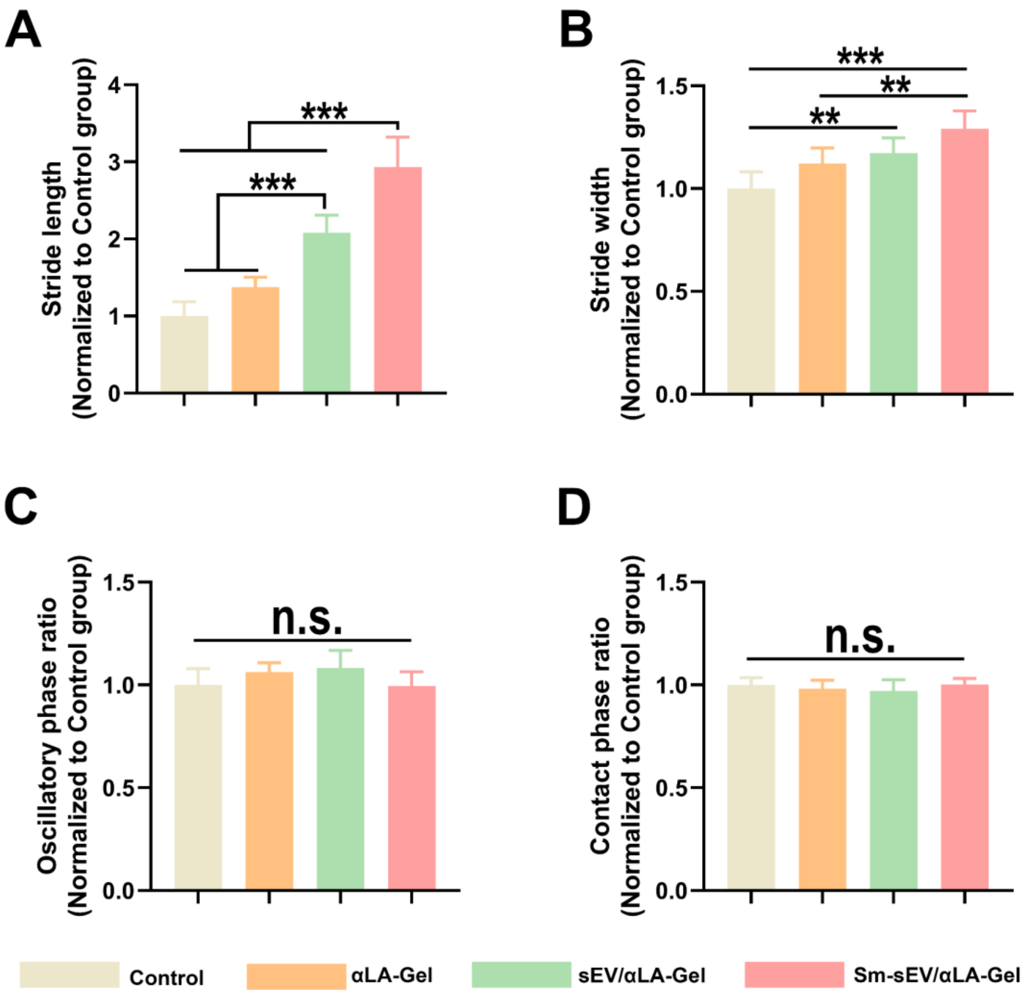

Figure S13. Kinematic gait analysis at 2 weeks post-surgery. (A) Stride length, (B) stride width, (C) oscillatory phase ratio, and (D) contact phase ratio in Control, αLA-Gel, sEV/αLA-Gel, and Sm-sEV/αLA-Gel groups. Data are normalized to the Control group. *p < 0.05, **p < 0.01, ***p < 0.001. n.s., not significant.

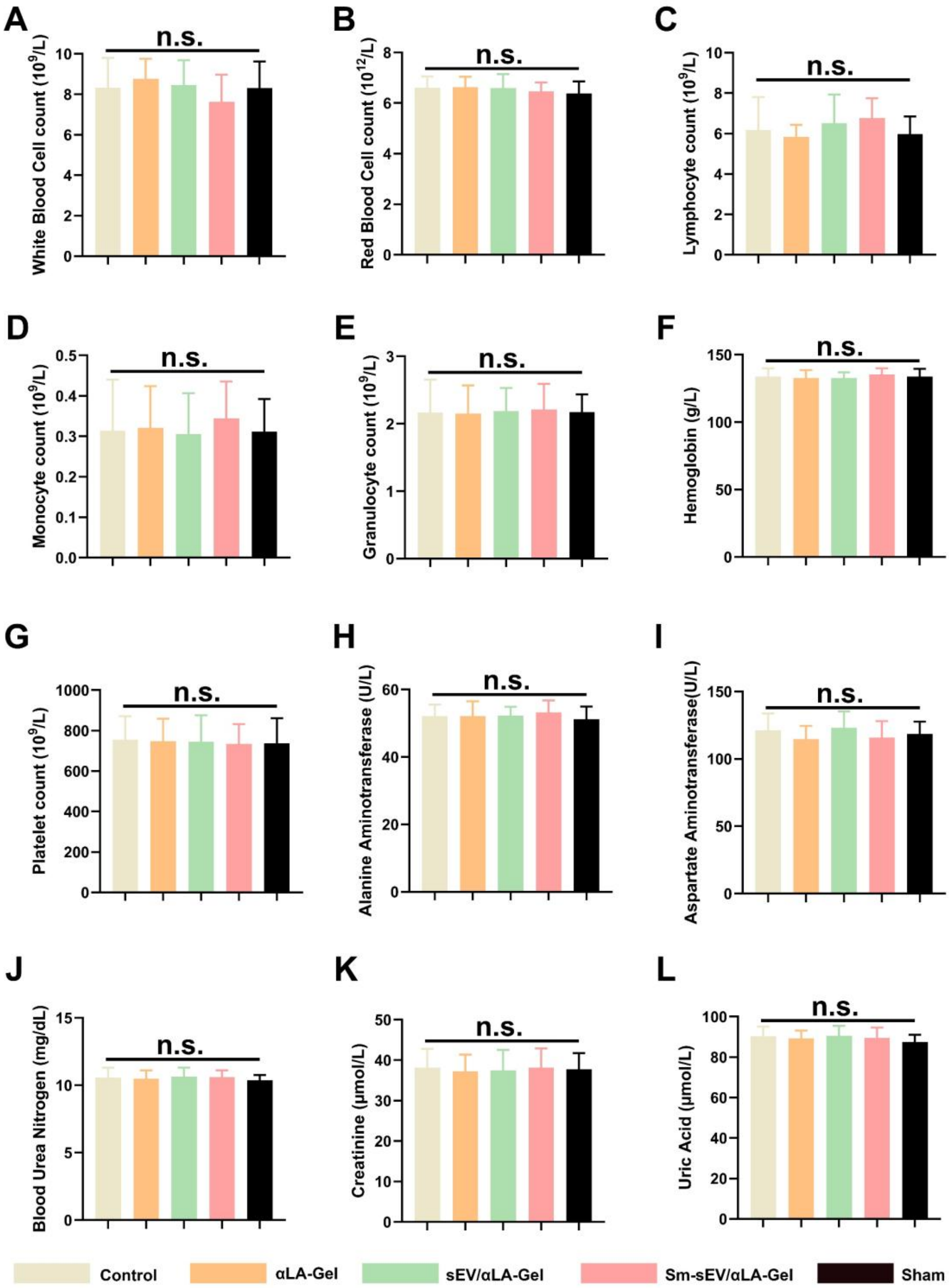

Figure S14. Hematological and biochemical evaluation of systemic biosafety. (A–G) Routine blood parameters, including white blood cell count, red blood cell count, lymphocyte count, monocyte count, granulocyte count, hemoglobin, and platelet count. (H–L) Serum biochemical indicators, including alanine aminotransferase, aspartate aminotransferase, blood urea nitrogen, creatinine, and uric acid. n.s., not significant.

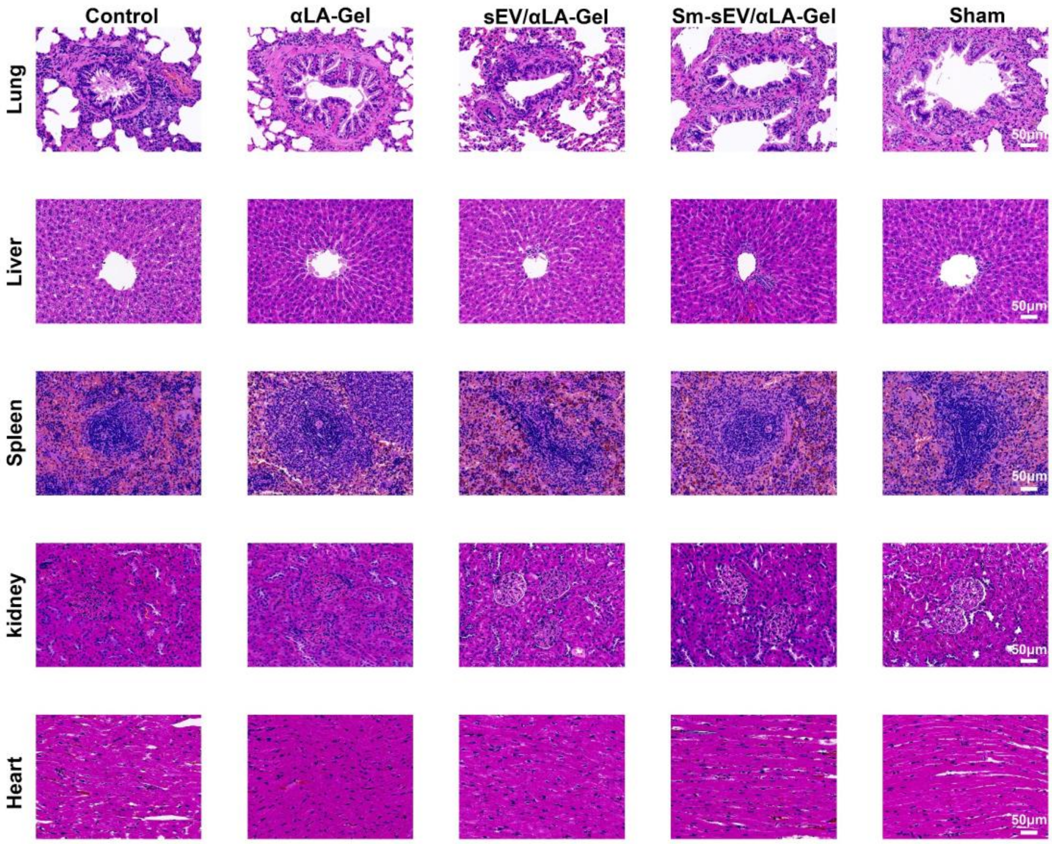

Figure S15. Histological evaluation of major organs after treatment. Representative H&E staining images of the lung, liver, spleen, kidney, and heart from Control, αLA-Gel, sEV/αLA-Gel, Sm-sEV/αLA-Gel, and Sham groups.

Table S1. Information on the primary antibodies used in this study.

Antibody Name	Company	Catalog Number
CD68	Santa Cruz	sc-20060
iNOS	Abcam	ab178945
CD206	Abcam	ab64693
p16	Proteintech	60626-1-Ig
p21	Proteintech	10355-1-AP
p21	Proteintech	28248-1-AP
γ-H2AX	Proteintech	68888-1-Ig
COL I	Servicebio	GB124197-100
OCN	Servicebio	23418-1-AP
cGAS	Servicebio	GB150056
STING	Servicebio	GB150063
p-IRF3	Servicebio	29528-1-AP
IRF3	Servicebio	GB11368
p-Iκβα	Servicebio	GB15212
Iκβα	Servicebio	GB13212
p-p65	Servicebio	GB113882
p65	Servicebio	GB11997
β-actin	Proteintech	66009-1-Ig
Anti-TNF alpha	Abcam	ab307164
IL-10	Proteintech	60269-1-Ig
BMP2	Servicebio	GB12252
Runx2	Proteintech	20700-1-AP
COL II	Proteintech	28459-1-AP

COL III	Servicebio	GB111629
CD9	Abcam	ab263019
CD81	Abcam	ab109201
HSP70	Abcam	ab181606
Calnexin	Abcam	ab133615

Table S2. Primer sequences for RT-qPCR

Gene	Species		Primer sequence
GAPDH	Homo sapiens	Forward	GGAAGCTTGTCATCAATGGAAATC
		Reverse	TGATGACCCTTTTGGCTCCC
p16	Homo sapiens	Forward	ATGTCGCACGGTACCTGC
		Reverse	AATCGGGGATGTCTGAGGGA
p21	Homo sapiens	Forward	GCGACTGTGATGCGCTAATG
		Reverse	GAAGGTAGAGCTTGGGCAGG
γ-H2AX	Homo sapiens	Forward	ATGTCGGGCCGCGGCAAG
		Reverse	TTAGTACTCCTGGGAGGCCTG
IL-1β	Homo sapiens	Forward	ATGGCAGAGTACCTGAGCTC
		Reverse	TTAGGAAGACACAAATGCATGGTG
TNF	Homo sapiens	Forward	CTTCCAGCTGGAGAAGGGTG
		Reverse	CCCAAAGTAGACCTGCCCAG
IL-6	Homo sapiens	Forward	AGTGAGGAACAAGCCAGAGC
		Reverse	GGTCAGGGGTGGTTATTGCA
Arg-1	Homo sapiens	Forward	AAGATTCCCGCATGTGCAGG
		Reverse	GTCCACGTCTCTCAAGCCAA
IL-1ra	Homo sapiens	Forward	GATGTGCCTGTCCTGTGTCA
		Reverse	ACTCAAAACTGGTGGTGGGG
IL-10	Homo sapiens	Forward	AAGACCCAGACATCAAGGCG
		Reverse	AGGCATTCTTCACCTGCTCC
ALP	Homo sapiens	Forward	AACATCAGGGACATTGACGTG
		Reverse	GTATCTCGGTTTGAAGCTCT
COL I	Homo sapiens	Forward	CCCCTGGAAAGAATGGAGATGA
		Reverse	CATCCAAACCACTGAAACCTCTG
RUNX2	Homo sapiens	Forward	GTGGACGAGGCAAGAGTTTCA
		Reverse	TCTGTCTGTGCCTTCTGGGTT
OCN	Homo sapiens	Forward	TCACACTCCTCGCCCTATTG
		Reverse	CTCCTGAAAGCCGATGTGGT

OPN	Homo sapiens	Forward	CGAAGTTTTCACTCCAGTTGTCC
		Reverse	AGGTGATGTCCTCGTCTGTAGC
CXCL10	Homo sapiens	Forward	TGCCATTCTGATTTGCTGCC
		Reverse	GCTGATGCAGGTACAGCGTA
IFNB1	Homo sapiens	Forward	TGCAGCAGTTTCCAGAAGGAG
		Reverse	AGTCTCATTCCAGCCAGTGC
ISG15	Homo sapiens	Forward	ACCTGACGGTGAAGATGCTG
		Reverse	ATCTTCTGGGTGATCTGCGC
NFKBIA	Homo sapiens	Forward	TACACCTTGCCTGTGAGCAG
		Reverse	AGCACCCAAGGACACCAAAA
CCL2	Homo sapiens	Forward	GACCATTGTGGCCAAGGAGA
		Reverse	TTGGGTTGCTTGTCCAGGT
PTGS2	Homo sapiens	Forward	ATGATTGCCCGACTCCCTTG
		Reverse	CATGTTTGAGCCCTGGGGAT
GAPDH	Rat	Forward	CTGGAGAAACCTGCCAAGTATG
		Reverse	GGTGGAAGAATGGGAGTTGCT
p16	Rat	Forward	CTCCTTGGCTTCACTTCTGG
		Reverse	CTCCCTCCCTCTGCTAACCT
p21	Rat	Forward	TTGTGATATGTACCAGCCACAG
		Reverse	CCATGAGCGCATCGCAATC
γ-H2AX	Rat	Forward	TGGAAAGGGTCAGGGAACG
		Reverse	GACTTGTGCTGGTATCTGGGTG

Table S3. Histological grading criteria for tendon maturation at the bone-tendon interface

Parameter	1 point	2 points	3 points	4 points
Cell density	High cell concentration	Moderate cellularity	Mild cellularity	Sparse cellularity
Tenocyte-like cell proportion	<25%	25-50%	50-75%	>75%
Cell alignment	<25% aligned	25-50% aligned	50-75% aligned	>75% aligned
Collagen fiber orientation	<25% parallel	25-50% parallel	50-75% parallel	>75% parallel
Vascularity	Abundant vessels	Moderate vascularity	Mild vascularity	Minimal vascularity
Large-diameter fiber content	<25%	25-50%	50-75%	>75%
Bone-tendon insertion organization	Disorganized	Mildly structured	Moderately structured	Well-organized
